# Hydrological projections in the upper reaches of the Yangtze River Basin from 2020 to 2050

**DOI:** 10.1038/s41598-021-88135-5

**Published:** 2021-05-06

**Authors:** Ya Huang, Weihua Xiao, Baodeng Hou, Yuyan Zhou, Guibing Hou, Ling Yi, Hao Cui

**Affiliations:** 1 State Key Laboratory of Simulation and Regulation of Water Cycle in River Catchment, China Institute of Water Resources and Hydropower Research, Beijing, 100038 China; 2 College of Oceanography, Hohai University, Nanjing, 210098 China; 3 China Water Resources Pearl River Planning, Surveying & Designing Co., Ltd., Zhanyi Road 19#, Guangzhou, 510610 China; 4 Global Institute for Water Security, University of Saskatchewan, Saskatoon, SK Canada

**Keywords:** Climate change, Hydrology

## Abstract

Understanding the impact of climate change on runoff is essential for effective water resource management and planning. In this study, the regional climate model (RCM) RegCM4.5 was used to dynamically downscale near-future climate projections from two global climate models to a 50-km horizontal resolution over the upper reaches of the Yangtze River (UYRB). Based on the bias-corrected climate projection results, the impacts of climate change on mid-twenty-first century precipitation and temperature in the UYRB were assessed. Then, through the coupling of a large-scale hydrological model with RegCM4.5, the impacts of climate change on river flows at the outlets of the UYRB were assessed. According to the projections, the eastern UYRB will tend to be warm-dry in the near-future relative to the reference period, whereas the western UYRB will tend to be warm-humid. Precipitation will decreases at a rate of 19.05–19.25 mm/10 a, and the multiyear average annual precipitation will vary between − 0.5 and 0.5 mm/day. Temperature is projected to increases significantly at a rate of 0.38–0.52 °C/10 a, and the projected multiyear average air temperature increase is approximately 1.3–1.5 ℃. The contribution of snowmelt runoff to the annual runoff in the UYBR is only approximately 4%, whereas that to the spring runoff is approximately 9.2%. Affected by climate warming, the annual average snowmelt runoff in the basin will be reduced by 36–39%, whereas the total annual runoff will be reduced by 4.1–5%, and the extreme runoff will be slightly reduced. Areas of projected decreased runoff depth are mainly concentrated in the southeast region of the basin. The decrease in precipitation is driving this decrease in the southeast, whereas the decreased runoff depth in the northwest is mainly driven by the increase in evaporation.

## Introduction

In the past few decades, the significant increase in temperature has led to an increase in the maximum amount of water vapor that can be carried by the atmosphere, which has affected the spatial and temporal distribution characteristics of precipitation^1–5^. Higher temperature also causes higher rates of surface drying and evaporation, thereby increasing the duration and intensity of droughts^6^. Many regions of the world can easily cope with moderate changes in the average climate and can even benefit from changing climate^7^. However, most of the destructive effects of floods, droughts or other disasters are the result of extreme weather and climate events, which are likely to occur more frequently on a global scale^8^ and have indirect and direct impacts on natural vegetation, urban construction, farming, energy generation, water resources and the environment^9–12^, resulting in considerable economic losses^13^. Accurate understanding and projections of the spatiotemporal characteristics of water resource changes in a basin caused by changes in precipitation are essential for effectively managing regional water resources, responding to various risks related to climate change, and formulating appropriate climate change adaptation and mitigation measures^14^.

The Yangtze River, the longest river in China, provides precious water resources for the Yangtze River Basin (YRB) and supports the livelihoods of millions of people. Due to the influences of the East Asian summer monsoon and the South Asian summer monsoon, the YRB exhibits complex and unique precipitation patterns and a unique regional climate^15,16^, and is a flood-prone area^17^. The upper reaches of the YRB (UYRB) accounts for approximately 59% of the YRB, and the multiyear average annual runoff accounts for approximately 46% of the YRB. Climate change has led to changes in the hydrological conditions in the UYRB, which have affected the amount of available water resources and the socioeconomic development and ecological environment of the middle and lower reaches of the YRB. For this reason, the impact of climate change on water resources in the UYRB and the future response of water resources in the basin to climate change have received widespread attention. A large number of observations and projection studies suggest that climate change has accelerated the hydrological cycle of the YRB, reduced the snow cover in the basin and increased extreme runoff. Previous studies have mainly used climate conditions predicted by global climate models (GCMs) to drive hydrological models to study the responses of UYRB hydrology to climate change^18–26^. However, few studies have investigated the hydrological changes in the UYRB under climate change using a coupling model based on the RegCM4 and variable infiltration capacity (VIC) models^27–29^.

RegCM has been applied to the UYRB as an effective tool for assessing and projecting hydroclimatic conditions^27–31^, but it remains challenging to accurately assess and project climate changes in the basin. Gao et al.^31^ found that, according to a coupling model of RegCM2 and CSIRO R21L9, precipitation in most regions of the YRB will increase in the future. Cao et al.^30^ found that, based on RegCM3 forcing by the FvGCM CCM3 under the SRES A2 scenario, summer precipitation in most areas of the YRB will decrease by the late twenty-first century. Gu et al.^29^ used the ECHAM5 results under the SRES A1B scenario to drive RegCM4 to project precipitation in the YRB to the end of the twenty-first century and found that precipitation in the north and south of the basin will increase and decrease, respectively. Lu et al.^28^ used the HadGEM2-ES under three representative concentration pathways (RCPs) scenarios (2.6, 4.5, and 8.5) to drive RegCM4 to project runoff in the source region of YRB for 2041–2060 and found that snowmelt runoff would become more important with increase of 17.5% and 18.3%, respectively, under RCP2.6 and RCP4.5 but decrease of 15.0% under RCP8.5. In general, the above results indicate that the total precipitation and probability of heavy precipitation in the YRB will significantly increase by the end of this century. However, investigations of near-future responses of hydroclimatic processes of the UYRB to global climate change are very limited.

The purpose of the current study was to evaluate the characteristics of changes in runoff in the UYRB under near-future climate change. To this end, the historical and future projection results provided by CSIRO-MK3.6.0 and MPI-ESM-MR were used to conduct a 50-km resolution dynamic downscaling experiment to estimate the characteristics of temperature and precipitation change in the UYRB under two RCPs (4.5 and 8.5) in the mid-twenty-first century. In addition, the quantile mapping method based on the mixed distribution was used to correct the bias of meteorological elements from the dynamic downscaling output of the regional climate model (RCM). Before using the VIC model, the generalized likelihood uncertainty estimation (GLUE) method was used to measure the uncertainty of the parameters of the VIC model. The corrected meteorological elements were used to drive the VIC hydrological model to analyze the impacts of near-future climate change on runoff in the UYRB. This study has certain reference significance for deepening the understanding of runoff change characteristics and water resource management in the UYRB under the background of global warming and provides a scientific basis for further development of adaptive measures.

## Models and data

### The climate model

#### Experimental design and model configuration

**Figure 1:**
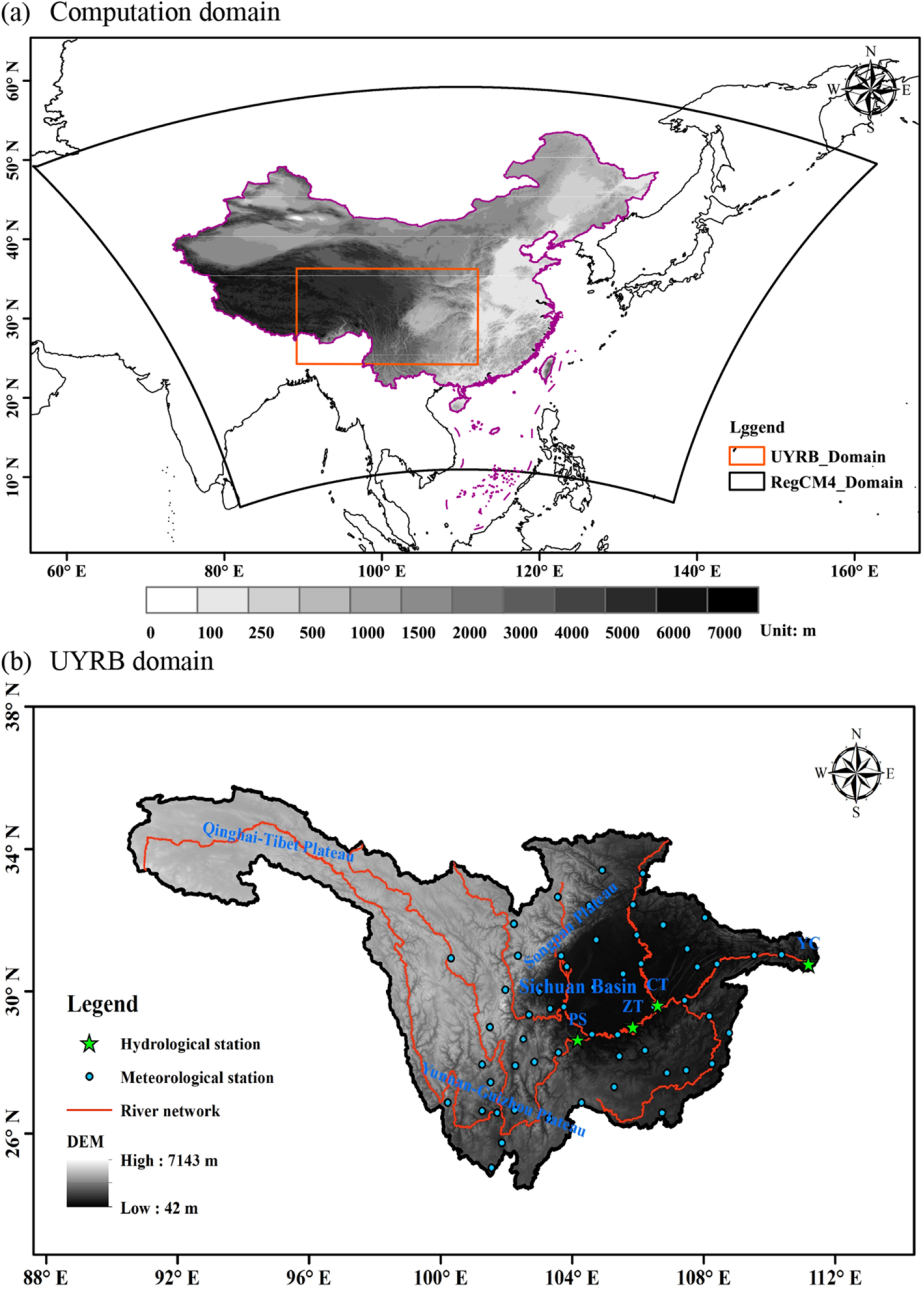
** (a)** Computation domain and topography (m) of RegCM4; (**b)** the UYRB domain and topography (m). The figure was generated by Arcmap 10.6 (https://www.esri.com).

**Figure 2:**
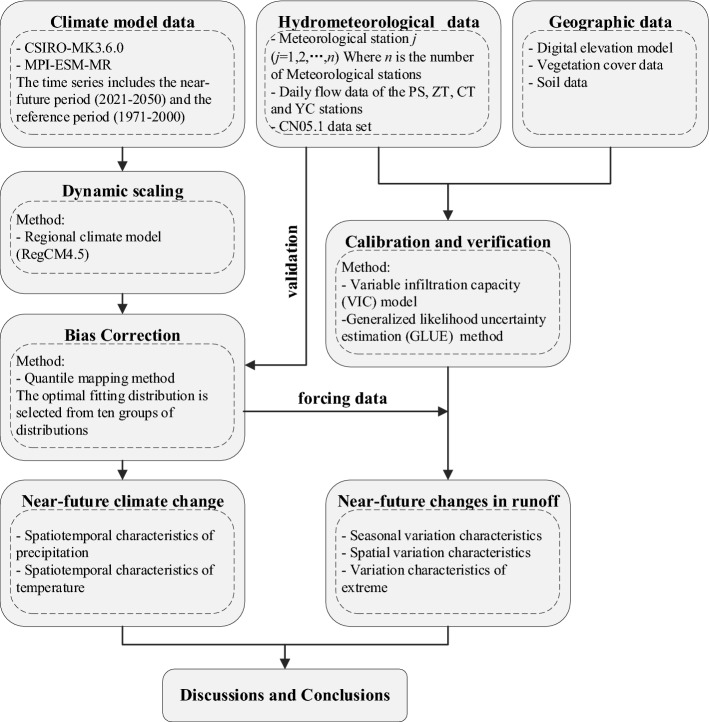
Modelling flowchart of this study. The figure was generated by Visio 2019 (https://www.microsoft.com/).

RegCM4.5 is an RCM developed by the Abdus Salam International Center for Theoretical Physics^32^ and has been extensively applied in multi-decadal climate change simulations in China^33,34^. Because the RegCM4.5 scheme configured by Gao et al.^33^ exhibits good simulation performance in China, it was adopted in this study. As shown in Fig. 1a, in this study, only the UYRB from East Asia was intercepted for analysis^35^. The topography and river system in the UYRB were shown in Fig. 1b. The radiation scheme used was the NCAR CCM3 scheme^36^. The cumulus convection scheme and planetary boundary layer scheme used in the current study were the Emanuel^37^ and Holtslag^38^ schemes, respectively. The Zeng sea surface flux parameterization scheme was used^39^. Details on the model parameter configuration are presented in Table 1. The processing of and analysis procedures for the various data sets used in this study are shown in Fig. 2.

**Table 1:** The RegCM4 model configuration used in this study.

Contents	Description
Domain	50 km horizontal resolution Central Lat. and Lon.: 35°N, 115°E 200 (Lon) × 130 (Lat)
Vertical layers (top)	18 vertical sigma levels (1 hPa)
PBL scheme	Holtslag
Cumulus parameterization scheme	Emanuel
Land surface model	NCAR CLM3.5
Short-/longwave radiation scheme	NCAR CCM3
Boundary data	CSIRO-MK3.6.0, MPI-ESM-MR
Simulation period	Jan. 1970-Dec. 2000; Jan. 2020-Dec. 2050
Analysis period	Jan. 1971-Dec. 2000; Jan. 2021-Dec. 2050

#### Correction of the RegCM4.5 outputs

The commonly used RCM bias correction methods are the Delta method^40^, statistical multiple regression models^41,42^, K-nearest neighbor approach^43^, nearest-neighbor technique^44^, and quantile mapping^45–47^. Themeßl et al.^47^ compared the correction performance of the above methods on RCM output results and found that the quantile mapping (QM) method had the best performance. Moreover, Shin et al.^45^ found that the QM method based on the mixed distribution is better than the QM method based on the single distribution in correcting precipitation, especially extreme precipitation. Therefore, the QM method based on the mixture distribution was used to correct the precipitation, and the temperature was corrected by the QM method based on the GEV distribution^48^. Due to the obvious seasonal characteristics of the climate in the YRB, the RCM output was corrected month by month based on long-term observation data.

Some distributions that are widely applied for modeling extreme events in hydrometeorological and many other fields^45,49–51^, such as the gamma, exponential, generalized extreme value (GEV), Gumbel and generalized Pareto (GP) distributions were tested to evaluate the optimal fitting distribution of precipitation events in the UYRB. The cumulative distribution function (CDF) and probability density function (PDF) of the above distributions are shown in Table 2.

**Table 2:** PDF and CDF of the functions used in the study.

Function	PDF	CDF
Gamma	$f_{G} (x) = \frac{{\alpha^{ - \beta } }}{\Gamma (\beta )}x^{\beta - 1} \exp ( - \frac{x}{\alpha })$	$F_{G} (x) = \int_{0}^{x} {\frac{{\alpha^{ - \beta } }}{\Gamma (\beta )}x^{\beta - 1} \exp ( - \frac{x}{\alpha })dx}$
Exponential	$f_{E} (x) = \frac{1}{\alpha }\exp ( - \frac{x}{\alpha })$	$F_{E} (x) = 1{\text{ - exp}}( - \frac{x}{\alpha })$
GEV	$f_{V} (x) = \frac{1}{\alpha }(1 + \beta ( - \frac{x - \tau }{\alpha })^{ - 1/\beta - 1} )\exp ( - (1 + \beta (\frac{x - \tau }{\alpha })^{ - 1/\beta } ))$	$F_{V} (x) = \exp ( - (1 + \beta (\frac{x - \tau }{\alpha })^{ - 1/\beta } ))$
Gumbel	$f_{U} (x) = \frac{1}{\alpha }\exp ( - \frac{x - \tau }{\alpha })\exp ( - \exp ( - \frac{x - \tau }{\alpha }))$	$F_{U} (x) = 1 - \exp ( - \exp ( - \frac{x - \tau }{\alpha }))$
GP	$f_{P} (x) = \frac{1}{\alpha }(1 + \beta (\frac{x - \tau }{\alpha })^{ - 1/\beta - 1} )$	$F_{P} (x) = 1 - (1 + \beta (\frac{x - \tau }{\alpha })^{ - 1/\beta } )$

The definition of a mixture distribution is as follows:

1 $$ f(x) = \sum\limits_{i = 1}^{n} {\sigma_{i} f_{i} } (x;\delta_{i} ) $$

where $\delta_{i}$ is a parameter of the *i*th distributed component in the mixture distribution, and $\sigma_{i}$ is the weight of the *i*th distributed component. *n* is the number of mixtures applied. Based on the characteristics of the above five distributions, we present five mixture distributions: gamma-gamma (G-G), gamma-exponential (G-E), gamma-Gumbel (G-U), gamma-GEV (G-V) and gamma-GP (G-P). Their PDFs are as follows:

2 $$ f_{{G{ - }G}} (x;\alpha_{1} ,\beta_{1} ,\alpha_{2} ,\beta_{2} ) = \sigma f_{G} (x;\alpha_{1} ,\beta_{1} ) + (1 - \sigma )f_{G} (x;\alpha_{2} ,\beta_{2} ) $$

3 $$ f_{{G{ - }E}} (x;\alpha_{1} ,\beta_{1} ,\alpha_{2} ) = \sigma f_{G} (x;\alpha_{1} ,\beta_{1} ) + (1 - \sigma )f_{E} (x;\alpha_{2} ) $$

4 $$ f_{{G{ - }V}} (x;\alpha_{1} ,\beta_{1} ,\alpha_{2} ,\beta_{2} ,\gamma ) = \sigma f_{G} (x;\alpha_{1} ,\beta_{1} ) + (1 - \sigma )f_{V} (x;\alpha_{2} ,\beta_{2} ,\gamma ) $$

5 $$ f_{{G{ - }U}} (x;\alpha_{1} ,\beta_{1} ,\alpha_{2} ,\beta_{2} ) = \sigma f_{G} (x;\alpha_{1} ,\beta_{1} ) + (1 - \sigma )f_{U} (x;\alpha_{2} ,\beta_{2} ) $$

6 $$ f_{{G{ - }P}} (x;\alpha_{1} ,\beta_{1} ,\alpha_{2} ,\beta_{2} ,\gamma ) = \sigma f_{G} (x;\alpha_{1} ,\beta_{1} ) + (1 - \sigma )f_{P} (x;\alpha_{2} ,\beta_{2} ,\gamma ) $$

The simulated precipitation of the RCM usually produces a large number of invalid precipitation events. To be consistent with the observed precipitation events, we corrected the RCM precipitation events based on the historical period.

The simulated precipitation of the RCM usually generates a large number of invalid precipitation events. To be consistent with the observed precipitation events, we corrected the daily RCM precipitation data based on daily precipitation observation data of the reference period. The probability *P* that the observation of daily precipitation is zero is defined as follows:

7 $$ P_{0}^{obs} { = }\frac{{n_{0}^{obs} }}{{N^{obs} }} $$

where $N^{obs}$ is the total number of days during years and $n_{0}^{obs}$ is the number of dry days in $N^{obs}$. The zero part of the daily precipitation of the outputs is generally smaller than the portion of the observation, i.e., $P_{0}^{RCM} < P_{0}^{obs}$ for the reference period. Therefore, it is necessary to set the RegCM4.5 precipitation values to zero so that $P_{0}^{RCM} = P_{0}^{obs}$:

8 $$ x = \left\{ \begin{gathered} x_{RCM} \quad  \; \; x_{RCM} > \phi \hfill \\ 0 \quad \quad \quad x_{RCM} \le \phi \hfill \\ \end{gathered} \right. $$

where $\phi$ is the threshold corresponding to RCM when $P_{0}^{RCM} = P_{0}^{obs}$.

To rapidly obtain the parameters of the mixture distributions, a genetic algorithm was used to optimize the parameters. Three indices, the relative error (*BIAS*), correlation coefficient (*CORR*) and Nash–Sutcliffe efficiency coefficient (*NSE*), were selected as the genetic algorithm optimization objective functions, and the same calculation weight was applied. The formulas of the three indices are as follows:

9 $$ BIAS = \frac{{\overline{P} - \overline{O}}}{{\overline{O}}}{{ \times 100\% }} $$

10 $$ CORR = \frac{{\sum\limits_{i = 1}^{N} {\left( {P_{i} - \overline{P}} \right)\left( {O_{i} - \overline{O}} \right)} }}{{\left[ {\sum\limits_{i = 1}^{N} {(P_{i} - \overline{P})^{0.5} } } \right]\left[ {\sum\limits_{i = 1}^{N} {(O_{i} - \overline{O})^{0.5} } } \right]}} $$

11 $$ NSE = 1 - \frac{{\sum\limits_{i = 1}^{N} {\left( {P_{i} - O_{i} } \right)^{2} } }}{{\sum\limits_{i = 1}^{N} {\left( {O_{i} - \overline{O}} \right)^{2} } }} $$

where $P_{i}$ and $O_{i}$ are the values of the *i*-th period in the fitting and observation, respectively; $\overline{P}$ and $\overline{O}$ are the average values of the fitting and observation, respectively; and *N* is the total number of samples.

### The large-scale hydrological model

The VIC hydrological model can be used to simultaneously simulate the energy balance and the water balance of the ground surface^52,53^. The model has been widely used in global and regional streamflow studies^54–59^.

#### Uncertainty of the VIC model

In the study, the GLUE method proposed by Beven et al.^60^ was used to measure the uncertainty of the parameters of the VIC model. The likelihood objective function (LOF) is mainly used to evaluate the fit between the observed and simulated results. The *NSE* and *BIAS*, two indices with the same weight, were taken into consideration. The LOF is defined as follows:

12 $$ OBJ = 0.5\left| {BIAS} \right| + 0.5(1 - NSE) $$

To obtain the uncertainty range of the VIC hydrological model at a certain confidence level, the LOF values of all parameter groups less than 0.5 were normalized and sorted. To quantify the uncertainty level of the VIC hydrological model, three commonly used evaluation indices were selected for uncertainty analysis. These indices are defined as follows^61^:

13 $$ {\text{Containing ratio: }}\,\left( {CR} \right)CR = \frac{{n_{{Q_{in} }} }}{n} \times 100\% $$

where $n$ is the total number of observed discharges and $n_{{Q_{in} }}$ is the number of observed discharges falling within the uncertainty intervals.

14 $$  {\text{Average bandwidth }}\,\left( B \right):\,B{ = }\frac{1}{n}\sum\limits_{i = 1}^{n} {\left( {Q_{i}^{upper} - Q_{i}^{lower} } \right)} $$

where $Q_{i}^{upper}$ and $Q_{i}^{lower}$ are the upper and lower values, respectively, at time *i*.

15 $$  {\text{Average asymmetry degree }}\,\left( S \right):\,S{ = }\frac{1}{n}\sum\limits_{i = 1}^{n} {\left| {\frac{{Q_{i}^{upper} - Q_{i}^{{}} }}{{Q_{i}^{upper} - Q_{i}^{lower} }} - 0.5} \right|} $$

where $Q_{i}^{{}}$ is the observed discharge corresponding to time *i*.

**Figure 3:**
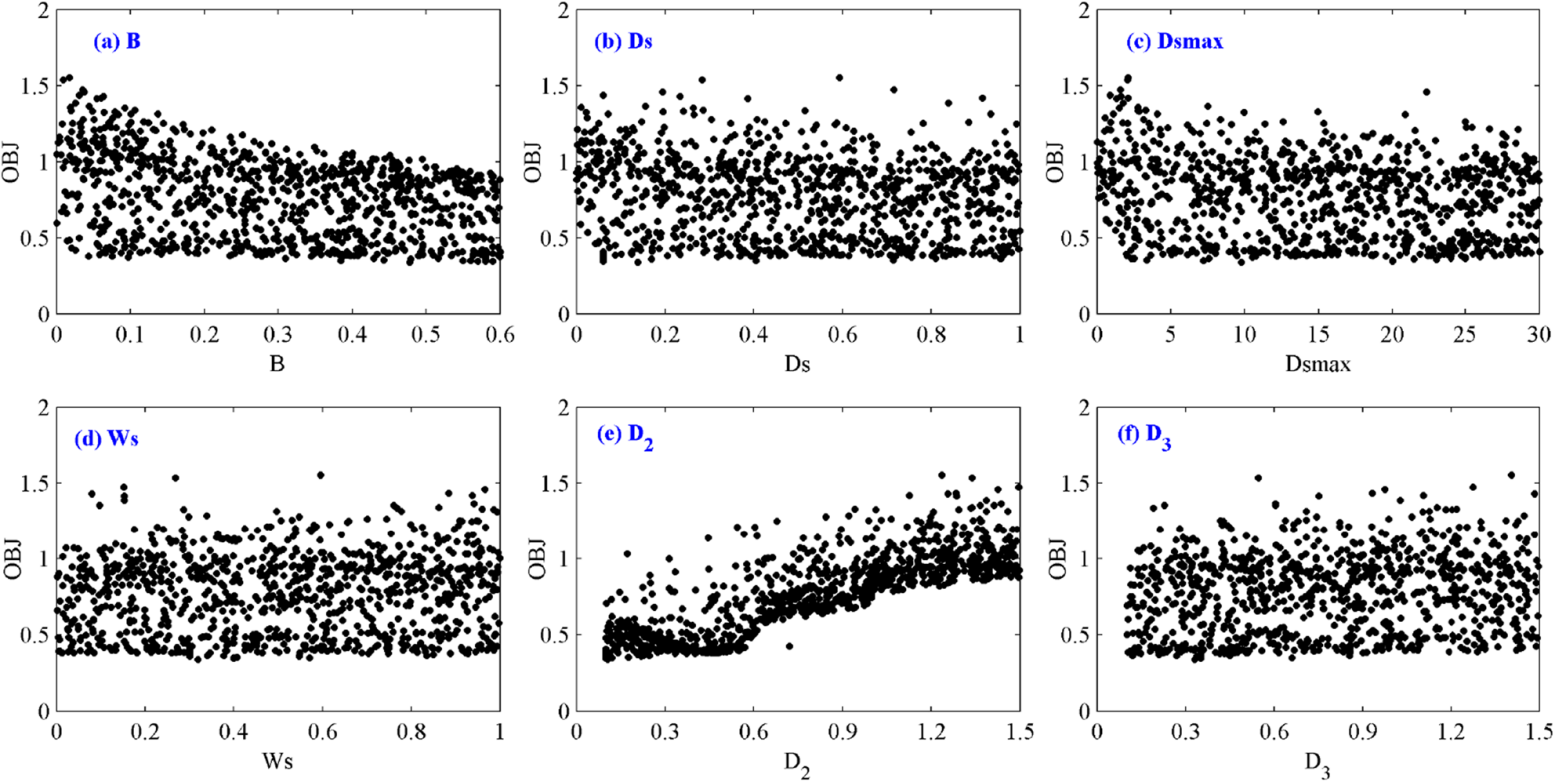
Scatter plots of likelihood objective function values for each parameter. The figure was generated by MATLAB2019a (https://www.mathworks.com/).

Figure 3 shows the scatter plots of the LOF for all parameters after the weighted calculation of four hydrological stations. In Fig. 3b–d, f, the scatter points of the LOF for the *D*_s_, *D*_smax_, *W*_s_ and *D*_3_ are approximately uniformly distributed and exhibit no obvious trend, indicating that these parameters are insensitive. For parameters *D*_2_ and *B*, the scatter points are unevenly distributed, which means that *D*_2_ and *B* are sensitive. The LOF decreases as *D*_2_ increases from 0.1 to 0.6 and increases as *D*_2_ increases from 0.6 to 1.5 (Fig. 3e). Parameter *B* ranges from 0 to 0.6, and the LOF decreases as *B* increases (Fig. 3a).

**Figure 4:**
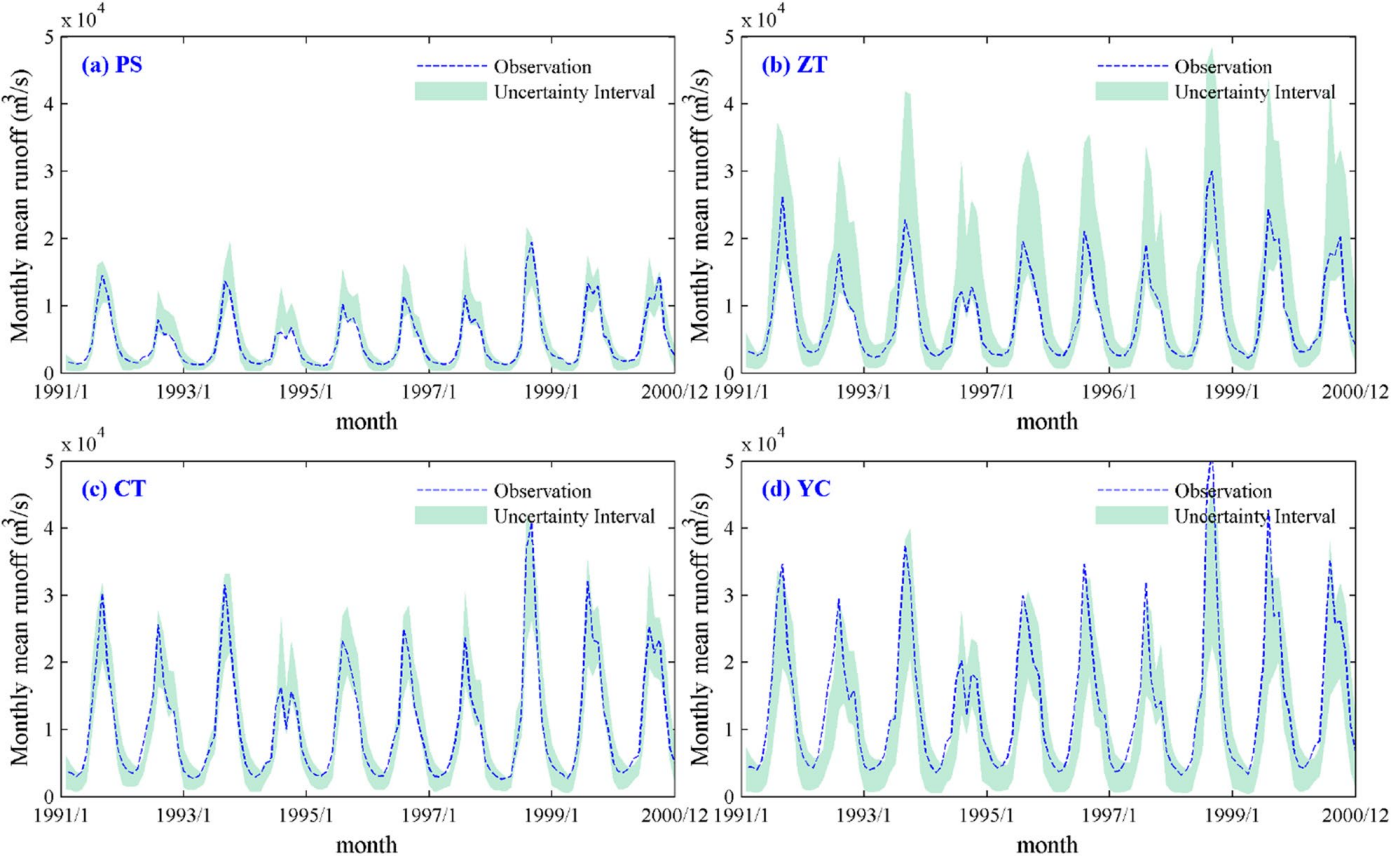
Uncertainty interval of monthly mean runoff at the 95% confidence level during the validation period. (**a** PS station; **b** ZT station; **c** CT station; **d** YC station). The figure was generated by MATLAB2019a (https://www.mathworks.com/).

As shown in Fig. 4a–d, the 95% confidence interval covers the observed flow of each station during the verification period, and only a few observed runoffs are outside the confidence interval, indicating that the VIC model is feasible for simulating runoff in the UYRB. The *CR* values at the Pingshan (PS), Zhutuo (ZT), Cuntan (CT) and Yichang (YC) stations are 77.5%, 98.3%, 79.2% and 75.3%, respectively. The *B* values are 3175, 10,736, 6201 and 9732, and the *S* values are 0.31, 0.25, 0.29 and 0.35, respectively (Table 3). In general, the VIC hydrological model has great uncertainty in low-flow and high-flow regions. The reason may be related to the prior distribution of the parameters. In addition, due to the complexity of the VIC hydrological model structure, there are a large number of optional parameter groups in the model; however, only 1,000 parameter groups were analyzed in the current study.

**Table 3:** Evaluation indices of the uncertainty interval during the validation period.

Indices	PS	ZT	CT	YC
*CR*	77.5	98.3	79.2	75.3
*B*	3175	10,736	6201	9732
*S*	0.31	0.25	0.29	0.35

#### Calibration and verification of the VIC hydrological model

**Figure 5:**
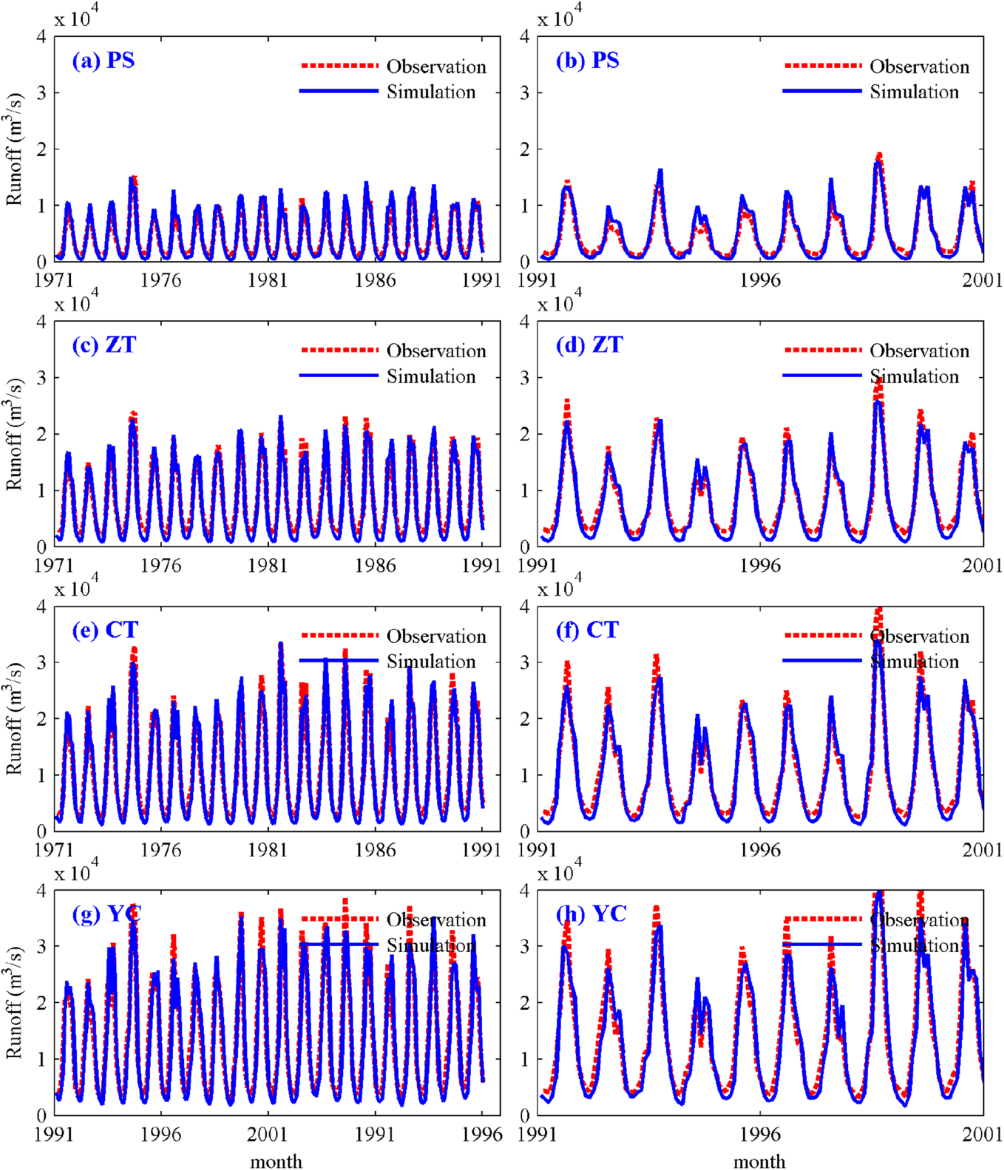
Comparisons of simulated and observed monthly mean runoff processes during calibration (**a,c,e,g**) and verification (**b,d,f,h**) periods. The figure was generated by MATLAB2019a (https://www.mathworks.com/).

In the process of model calibration and verification, the periods of calibration, verification and warm-up were set to 1971–1990, 1991–2000 and 1961–1970, respectively. The VIC model parameters were selected based on results described in the previous section. The detailed parameter configuration is shown in Table S1. As shown in Fig. 5a–h, the VIC model can simulate the runoff process and peak time of the PS, ZT, CT and YC stations, but the minimum and maximum values of discharge are underestimated. The *NSE*s of the respective PS, ZT, CT and YC stations are 0.90, 0.95, 0.96 and 0.93 during the calibration period and 0.92, 0.95, 0.95 and 0.92 during the verification period (see Table 4). The *BIAS* values of the respective PS, ZT, CT and YC stations are 1.41%, − 6.67%, − 2.88% and − 3.61% during the calibration period and 1.14%, − 7.88%, − 4.41% and − 5.86% during the verification period (see Table 4). The above results show that the VIC model has good simulation performance for the monthly average flow of the basin.

**Table 4:** Simulation performance of monthly mean runoff during the calibration and validation periods.

Station	Calibration period		Verification period	
	*NSE*	*BIAS* (%)	*NSE*	*BIAS* (%)
PS	0.90	1.41	0.92	1.14
ZT	0.95	− 6.67	0.95	− 7.88
CT	0.96	− 2.88	0.95	− 4.41
YC	0.93	− 3.61	0.92	− 5.87

To further analyze the simulation performance of the VIC hydrological model with respect to the runoff process in the UYRB, we analyzed the daily runoff process in a typical wet year (1981) and a typical dry year (1994). As shown in Fig. S1 (a-d), the VIC hydrological model performs well in simulating the daily runoff process in the UYRB. The coefficient of determination (*R*^2^) between the observed and simulated daily runoff at the PS, ZT, CT and YC stations reaches 0.9, 0.92, 0.91 and 0.91, respectively (Fig. S1). As shown in Fig. S2, the *NSE*s of the respective PS, ZT, CT and YC stations are 0.9, 0.93, 0.92 and 0.9 in the wet year (Fig. S2a, c, e, g) and 0.68, 0.85, 0.86 and 0.81 in the dry year (Fig. S2b, d, f, g). The *BIAS* values of the four stations are 13.52%, −0.45%, 0.32% and 0.33% in the wet year and −5.56%, −9.33%, −0.55% and −0.41% in the dry year (see Table S2). In general, the VIC hydrological model has good applicability in the UYRB, and its simulation performance in the wet year is slightly better than that in the dry year.

### Trend analysis

The nonparametric Mann–Kendall (MK) method^62,63^ was used to analyze the temporal trends of climatic factors, with significance evaluated at the 95% confidence level. The nonparametric MK method is considered a simple and effective way of conducting climate change analysis and has been extensively used in the analysis of hydrometeorological time series sets^50,64^. The MK statistical test is given as follow:

16 $$ Z{ = }\left\{ \begin{aligned} & \frac{S - 1}{{\sqrt {Var\left( S \right)} }} \, \quad \quad \; \; S > 0 \hfill \\ & 0 \,\quad \quad\quad \quad \quad \quad S = 0 \hfill \\ & \frac{S + 1}{{\sqrt {Var\left( S \right)} }} \, \quad \quad \; S < 0 \hfill \\ \end{aligned} \right. \, $$

where statistic *S* can be calculated as:

17 $$ S = \sum\limits_{i = 1}^{n - 1} {\sum\limits_{j = i + 1}^{n} {{\text{sgn}} \left( {x_{j} - x_{i} } \right)} } $$

where $x_{i}$ and $x_{j}$ are the observations at the *i*th and *j*th moments, respectively, and *n* is the length of the series. When $x_{i}$-$x_{j}$ is greater than, equal to or less than 0, ${\text{sgn}} \left( {x_{i} - x_{j} } \right)$ equals 1, 0, or − 1, respectively.

The statistic Z can be used as a measure of a trend. Z > 0 and Z < 0 indicate increasing and decreasing trends, respectively. A larger |Z| value indicates a more significant trend. In this study, significance level was evaluated at the 0.05 level, which mean that Z > 1.96 and Z <  − 1.96 indicated significant increasing and decreasing trends, respectively.

Since the autocorrelation of a time series may affect the accuracy of trend analysis, the method developed by Yue and Wang^65^ was used to eliminate the possible autocorrelation in the extreme precipitation data series for the UHRB. In addition, Sen’s slope^66^ was used to determine the degree of trend, as it can eliminate the impact of missing data or anomalies on the trend test. The slope is estimated by

18 $$\beta { = }Median\left[ {\frac{{\left( {x_{j} - x_{i} } \right)}}{{\left( {j - i} \right)}}} \right],\quad \forall j > i $$

where $\beta$ is the estimate of the slope of the trend and $x_{i}$ and $x_{j}$ are the observations at the *i*th and *j*th moments, respectively.

### Applied data

#### Meteorological observation data

The meteorological observation data used in the UYRB were extracted based on the CN05.1 data with a resolution of 0.5° developed by Wu and Gao^67^, which include 329 meteorological stations in the YRB. Forty-two sites with relatively high quality were selected for the performance evaluation of the bias correction, and the meteorological station information is shown in Table 5. The CN05.1 data contain all meteorological elements required by VIC hydrological models and have been extensively applied in simulation performance evaluation and the analysis of climate models^33^. The inverse distance weighted method was used to interpolate CN05.1 data into the computational grid center of the RegCM4.5 model and the VIC model.

**Table 5:** Meteorological stations used for the correction performance assessment.

ID	Station	Name	Latitude	Longitude	ID	Station	Name	Latitude	Longitude
1	52908	Wudaoliang	35.3°	93.6°	22	56565	Yanyuan	27.4°	101.6°
2	56004	Tuotuohe	34°	92.6°	23	56571	Liangshan	27.9°	102.3°
3	56029	Yushu	32.9°	96.7°	24	56651	Lijiang	26.9°	100.2°
4	56034	Qingshuihe	33.9°	97.5°	25	56671	Huili	26.8°	102.3°
5	56144	Dege	31.8°	98.6°	26	56684	Huize	26.4°	103.2°
6	56146	Ganzi	31.6°	100°	27	56751	Dali	25.7°	100.2°
7	56167	Daofu	31°	101.2°	28	56778	Kunming	25°	102.7°
8	56172	Maerkang	31.9°	102.6°	29	57211	Ningqiang	32.7°	106.4°
9	56178	Xiaojin	31°	102.4°	30	57237	Wanyuan	32.1°	108.2°
10	56182	Songpan	32.6°	103.6°	31	57238	Zhenba	32.3°	108.1°
11	56188	Dujiangyan	31°	103.6°	32	57306	Langzhong	31.6°	106°
12	56385	Emeishan	29.5°	103.7°	33	57313	Bazhong	31.9°	106.8°
13	56386	Leshan	29.5°	103.8°	34	57348	Fengjie	31.1°	109.5°
14	56444	Deqin	28.8	98.8°	35	57355	Badong	31.1°	110.4°
15	56462	Jiulong	29°	101.6°	36	57405	Suining	30.5°	105.4°
16	56475	Yuexi	28.6°	102.6°	37	57411	Nanchong	30.8°	106.1°
17	56479	Shaojue	28.2°	103°	38	57445	Jianzhi	30.6°	109.6°
18	56485	Leibo	28.3°	103.6°	39	57461	Yichang	30.7°	111.1°
19	56492	Yibin	28.8°	104.5°	40	57502	Dazu	29.7°	105.7°
20	56543	Diqing	27.5°	100°	41	57517	Jiangjin	29.3°	106.3°
21	56548	Weixi	27.2°	99.5°	42	57608	Xuyong	28.2°	105.4°

#### Climate model data

The reference experimental and projected experimental results of CSIRO-MK3.6.0 and MPI-ESM-MR under the RCPs (4.5 and 8.5) were used as the initial and lateral boundary conditions for the study^68,69^. CSIRO-MK3.6.0 and MPI-ESM-MR were submitted by the Max Planck Institute of Germany and the Commonwealth Scientific and Industrial Research Organization of Australia, respectively, to the Coupled Model Intercomparison Project Phase 5 (CMIP5). The difference between the near-future period (2021–2050) and the reference period (1971–2000) under the RCPs (4.5 and 8.5) was considered to be the climate change in the UYRB.

#### VIC model forcing data

The hydrological data used for calibration and verification of the VIC hydrological model are the daily flow data of the PS, ZT, CT and YC stations from 1961 to 2000. The basic information of the hydrological station is shown in Table 6. The topography of the UYRB was defined from a digital elevation model (DEM) than can be downloaded from the website: http://www.gscloud.cn. The soil data were extracted from the global 5-min soil data set from the NOAA hydrological office (http://www.fao.org/soils-portal/en/). The vegetation cover data were obtained from the land cover classification data set with a 1 km resolution from the University of Maryland (http://www.landcover.org/data/landcover/data.shtml).

**Table 6:** Geographic location information of hydrological stations.

Station	Longitude	Latitude
PS	104.14°	28.64°
ZT	105.85°	29.02°
CT	106.57°	29.59°
YC	111.18°	30.77°

## Results

### Bias correction using the QM method

#### Correction performance for different distributions

To evaluate the performance of the QM method based on the mixture distribution for precipitation correction, observed precipitation data from 42 stations with good quality in the UYRB were applied. Table 7 shows the fitting performance statistical parameters of the QM method based on different distributions, such as the statistics of the Kolmogorov–Smirnov test (*D*), number of stations passing the Kolmogorov–Smirnov test at the 95% confidence level (*P*_95_), root mean square error (*RMSE*), mean relative error (*MRE*), sum of squares due to error (*SSE*) and *CORR*. According to the results in Table 7, the QM method based on mixed distribution has significantly better fitting performance regarding observed precipitation than that based on a single distribution. In the statistical results of the QM method based on the single distribution, few stations passed the significance test at the 95% confidence level, indicating that the single distribution fitting the observed precipitation in the UYRB is not applicable. The fitting performance of the mixed distribution for observed precipitation in the UYRB was significantly higher than that of the single distribution, especially G-G, G-E and G-V. The statistical results in Table 7 show that among all the mixed distributions, the G-V distribution achieved the best overall performance. Therefore, the G-V distribution was selected to correct precipitation data from the RegCM4.5 output.

**Table 7:** Statistical parameters of the fitting performance.

ID	Function	*D*	*P*_95_	*MRE*	*RMSE*	*SSE*	*CORR*
1	Gamma	0.32	23	20.59	6.87	1.66	1.00
2	Exponential	0.54	8	54.90	27.08	22.74	0.98
3	Gumbel	0.80	0	164.92	43.75	63.23	0.94
4	GP	0.51	0	− 49.63	15.28	8.45	0.99
5	GEV	0.38	14	9.32	13.44	6.01	0.99
6	G-G	0.18	38	1.17	1.39	0.16	1.00
7	G-E	0.19	40	− 1.26	1.35	0.18	1.00
8	G-U	0.37	13	8.10	3.17	0.38	1.00
9	G-V	0.17	39	− 1.07	1.29	0.14	1.00
10	G-P	0.23	16	− 0.66	2.04	0.46	1.00

#### Bias correction using the G-V distribution

**Figure 6:**
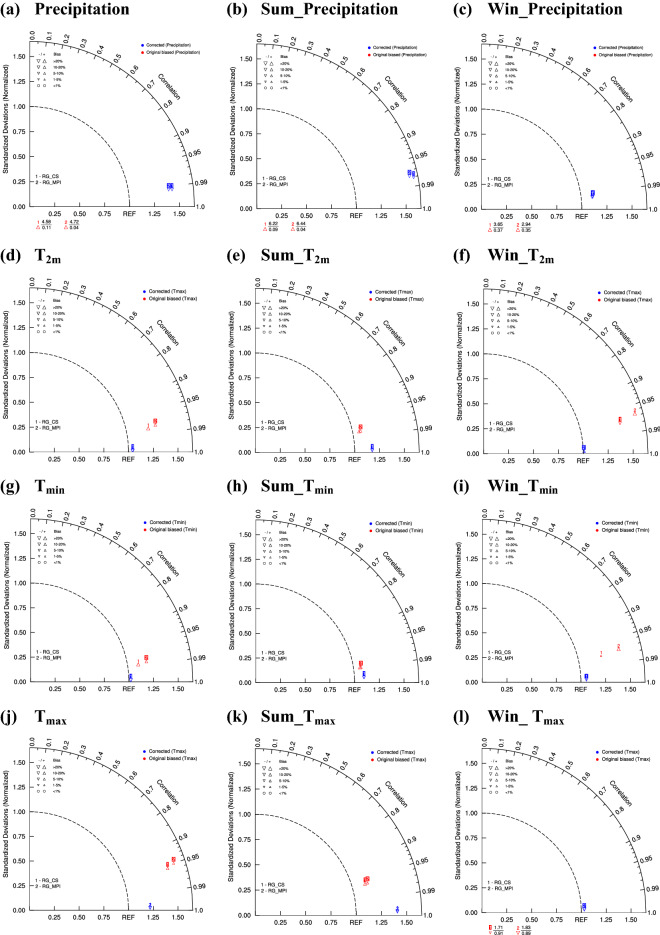
Taylor diagrams of precipitation **(a–c)**, T_2m_ **(d–f)**, T_min_ **(g–i)** and T_max_ **(j–l)** before and after the revision from R_CS and R_MPI for the reference period. (annual, left panels; summer, middle panels; winter, right panels). The figure was prepared using The NCAR Command Language version 6.5.0. (https://doi.org/10.5065/D6WD3XH5).

Figure 6 shows the Taylor diagrams of various meteorological elements in the UYRB before and after the revision from the CSIRO-MK3.6.0 and MPI-ESM-MR downscaling results (defined as R_CS and R_MPI, respectively) for the reference period. As shown in Fig. 6(a-c), poor performance was achieved in precipitation simulation in the UYRB from R_CS and R_MPI before the correction (marked in red). The spatial correlation coefficient of annual precipitation between simulated and observed precipitation was only approximately 0.1, whereas it can reach approximately 0.35 in winter, and the precipitation overestimation usually exceeded 20%. After the correction (marked in blue), the spatial correlation coefficient of annual precipitation between simulated and observed precipitation increased from 0.1 to 0.99, and the precipitation bias was reduced to approximately 10%.

As shown in Fig. 6d–l, the annual average air temperature (T_2m_), minimum temperature (T_min_), and maximum temperature (T_max_) were typically well simulated even before the correction (marked in red), and the spatial correlation coefficient of the air temperature between the simulated and observed values was usually approximately 0.95. The average annual and summer air temperatures had a warm bias of approximately 20% compared with an approximately 20% cold bias in winter. After the correction (marked in blue), the spatial correlation coefficient of the air temperature between the simulated and observed values increased from 0.95 to 0.99, and the bias of the air temperature was reduced from 20 to 10%.

**Figure 7:**
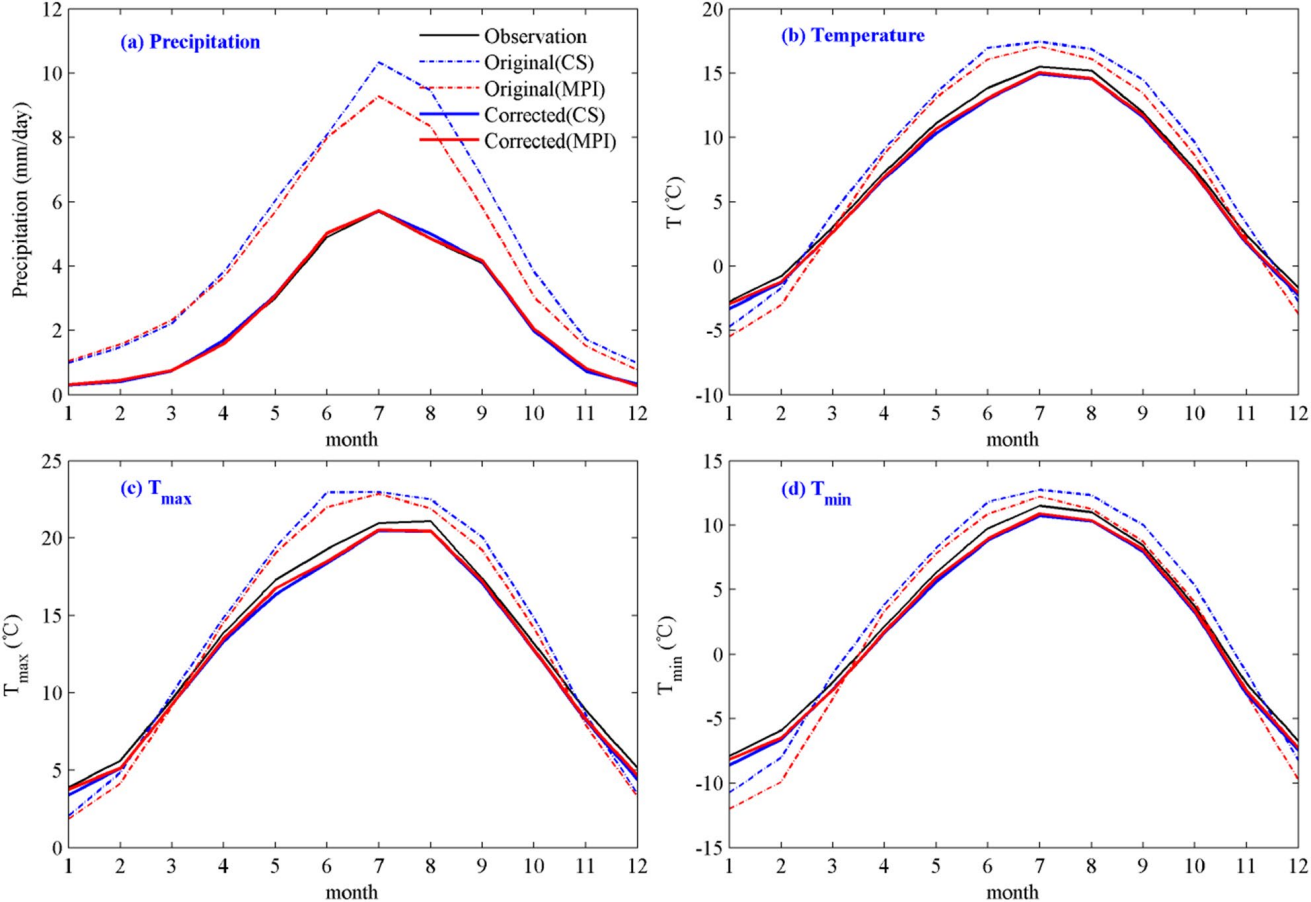
Annual cycles of precipitation **(a)**, T_2m_ **(b)**, T_max_ **(c)** and T_min_ **(d)** before and after the revision from R_CS and R_MPI. (The black line represents the observation; the blue and red dashed lines represent the result before the R_CS and R_MPI corrections, respectively; the blue and red lines represent the result after the R_CS and R_MPI corrections, respectively). The figure was generated by MATLAB2019a (https://www.mathworks.com/).

Figure 7 shows the annual cycles of precipitation, T_2m_, T_max_ and T_min_ before and after the revision from R_CS and R_MPI. As shown in Fig. 7a, the unrevised precipitation from R_CS and R_MPI was overestimated in the UYRB, whereas the revised precipitation was consistent with the observations. Similarly, compared with the observations, T_2m_, T_max_ and T_min_ before the revision exhibited some deviation in the annual cycle, among which warm biases mainly occurred in spring, summer and autumn, while cold biases occurred in winter (Fig. 7b–d). After the correction, the warm and cold biases were greatly reduced, and the annual cycle of temperature was consistent with the observation.

To further understand the performance of the correction using the QM method, Figs. S3-S6 (a-o) show the spatial distribution of annual mean precipitation, T_2m_, T_max_ and T_min_ in the UYRB before and after the revision from R_CS and R_MPI, and the corresponding results for summer and winter are also presented. After the revision, the precipitation biases that prevailed in the mountainous areas from western Sichuan to southern Qinghai and the southeastern region of the UYRB were significantly improved, and the warm bias of the Sichuan Basin and the cold bias of the source area of ​​the YRB were significantly improved (Fig. S3-S6). In short, the simulation performance regarding precipitation and air temperature from R_CS and R_MPI was greatly improved after correction using the QM method. From the next section onward, all meteorological elements referring to R_CS and R_MPI are corrected using the QM method.

### Near-future climate change projected by RegCM4.5

#### Near-future precipitation projected by RegCM4.5

**Figure 8:**
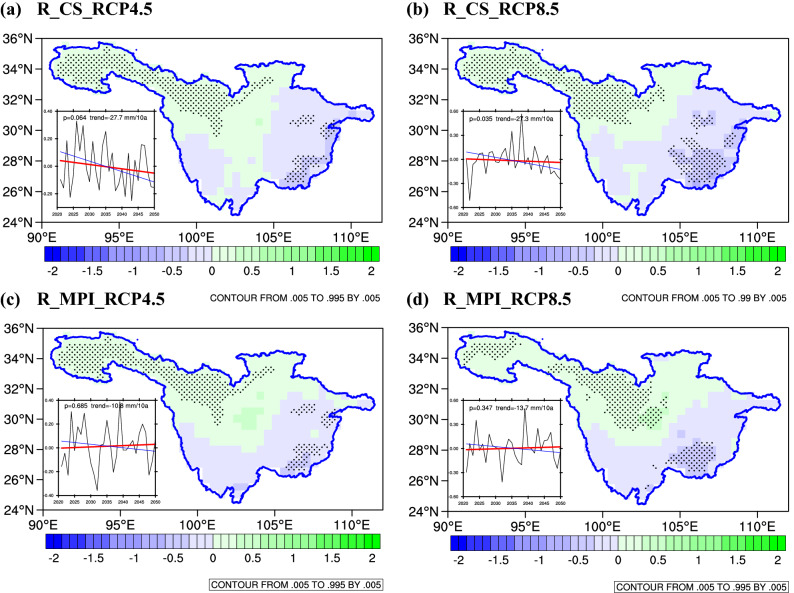
Multiyear average changes (unit: mm/day) in precipitation over the UYRB under the RCP4.5 and RCP8.5 scenarios compared to the reference period (1971–2000). The black dots denote differences that are statistically significant at a significance level of 95% based on Student’s t-test. The rectangle indicates the interannual variation trend of precipitation anomalies (unit: mm/day). The figure was prepared using The NCAR Command Language version 6.5.0. (https://doi.org/10.5065/D6WD3XH5).

Figure 8 shows the changes in the multiyear average precipitation under the RCPs (4.5 and 8.5) for R_CS (Fig. 8a,b) and R_MPI (Fig. 8c,d) in the mid-twenty-first century (defined as 2021–2050 minus 1971–2000). The block diagram shows the interannual variation trend in multiyear average precipitation anomaly. The black dotted shows the ​​changes that passed the significance test. In general, the multiyear average precipitation of the UYRB from R_CS and R_MPI during the 2021–2050 period exhibited an insignificant downward trend. Based on the spatial distribution of precipitation, there are obvious differences between the east and west of the UYRB. As shown in Fig. 8, the multiyear average precipitation in the Sichuan Basin increased significantly in the northwestern areas but decreased significantly in the southeast areas. Compared with the reference period, the multiyear average precipitation in the future will increase significantly by approximately 0.5 mm/day in the northwestern part of the basin and will decrease significantly by approximately 0.5 mm/day in the southeast of the basin.

Table 8 shows the variation of multiyear average precipitation in different periods under the RCPs (4.5 and 8.5) compared with the reference period. Under the RCPs (4.5 and 8.5), the changes in precipitation projected by R_CS and R_MPI in different periods were slightly different, but precipitation in both projections generally showed a trend of first increasing and then decreasing. In R_CS, the precipitation decrease was concentrated in 2031–2040 and was reduced by 0.048 mm/day and 0.088 mm/day under the RCP4.5 and RCP8.5, respectively. In R_MPI, the degree of precipitation decrease was smaller than that of R_CS, and the precipitation decrease was concentrated in 2040–2050. During this period, the precipitation decreases by 0.014 mm/day and 0.035 mm/day under the RCP4.5 and RCP8.5, respectively.

**Table 8:** Changes and trends of precipitation from R_CS and R_MPI under the RCP4.5 and RCP8.5 scenarios in different periods.

Periods	R_CS		R_MPI		Average	
	RCP4.5	RCP8.5	RCP4.5	RCP8.5	RCP4.5	RCP8.5
2021–2030	0.025	− 0.047	0.012	0.036	0.018	− 0.006
2031–2040	0.010	0.086	− 0.014	− 0.019	− 0.002	0.033
2041–2050	− 0.048	− 0.088	0.020	0.018	− 0.014	− 0.035
Mean	− 0.004	− 0.016	0.006	0.011	0.001	− 0.002
Trend (mm/10 a)	− 27.7	− 27.3*	− 10.8	− 13.7	− 19.25	− 19.05

*indicates a significant value at the 0.01 level.

**indicates a significant value at the 0.05 level.

#### Near-future temperature projected by RegCM4.5

**Figure 9:**
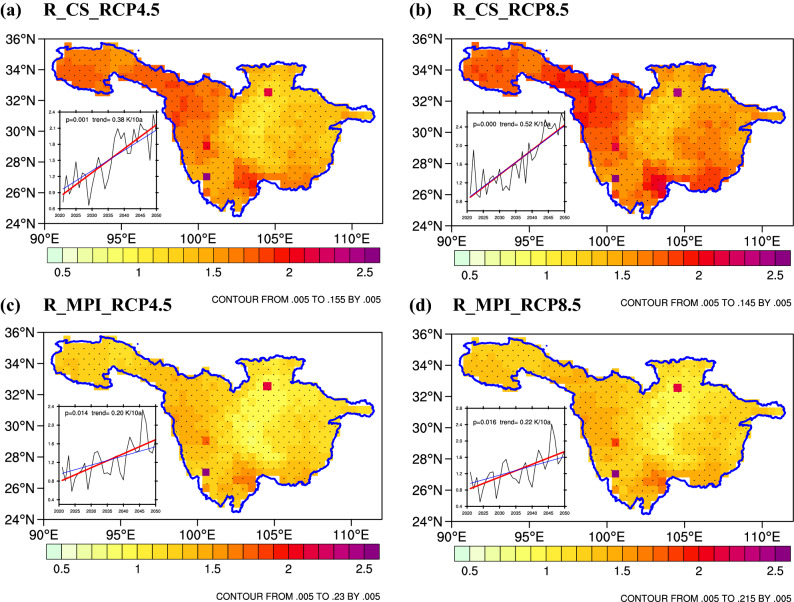
Multiyear average changes (unit: ℃) in T_2m_ over the UYRB under the RCP4.5 and RCP8.5 scenarios compared to the reference period (1971–2000). The black dots denote differences that are statistically significant at a significance level of 95% based on Student’s t-test. The rectangle indicates the interannual variation trend of air temperature anomalies (unit: ℃/10 a). The figure was prepared using The NCAR Command Language version 6.5.0. (https://doi.org/10.5065/D6WD3XH5).

Figure 9 and Figures S7-S8 show the changes in the multiyear average T_2m_ (Fig. 9a–d), T_max_ and T_min_ under the RCPs (4.5 and 8.5) for R_CS (Fig. 9a,b, S7a,b and S8a,b) and R_MPI (Fig. 9c,d, S7c,d and S8c,d) in the mid-twenty-first century (defined as 2021–2050 minus 1971–2000). In general, compared with the reference period, the multiyear average T_2m_ of the UYRB will increase by approximately 1–1.5 °C in the future, and the increasing trend will reach 0.29 °C/10 a and 0.37 °C/10 a under RCP4.5 and RCP8.5, respectively. In the future, the greater temperature increase will be mainly concentrated in the area from the Songpan Plateau to the east of the Qinghai-Tibet Plateau, where the T_max_ and T_min_ increases are usually above 2 °C. Table 9 and Tables S3-S4 show the variation of the multiyear average T_2m_, T_max_ and T_min_ in different periods under the RCPs (4.5 and 8.5) compared with the reference period. It can be seen from Table 9 that the T_2m_ increase is approximately 1 °C during the 2021–2030 period, while by the middle of the twenty-first century, the T_2m_ increase will reach approximately 1.5–2 °C.

**Table 9:** Changes and trends of T_2m_ from R_CS and R_MPI under the RCP4.5 and RCP8.5 scenarios in different periods.

Periods	R_CS		R_MPI		Average	
	RCP4.5	RCP8.5	RCP4.5	RCP8.5	RCP4.5	RCP8.5
2021–2030	1.046	1.247	0.967	0.952	1.007	1.099
2031–2040	1.567	1.446	1.141	1.208	1.354	1.327
2041–2050	1.937	2.305	1.660	1.688	1.798	1.997
Mean	1.516	1.666	1.256	1.283	1.386	1.474
Trend (℃/10 a)	0.38**	0.52**	0.20*	0.22*	0.29*	0.37*

*indicates a significant value at the 0.01 level.

**indicates a significant value at the 0.05 level.

In the future, the spatial changes of precipitation and temperature will be quite different between the western and eastern areas of the UYRB. On the whole, the eastern part of the basin shows a warm and dry trend, while the western part of the basin shows a warm and wet trend. Precipitation has strong interdecadal variation characteristics from 2021 to 2050, with a trend of first increasing and then decreasing, but the above trends are not significant. However, the temperature from 2021 to 2050 will continue to increase significantly, and the rate of warming will accelerate significantly.

### Near-future changes in runoff

#### Seasonal variation characteristics of runoff

**Figure 10:**
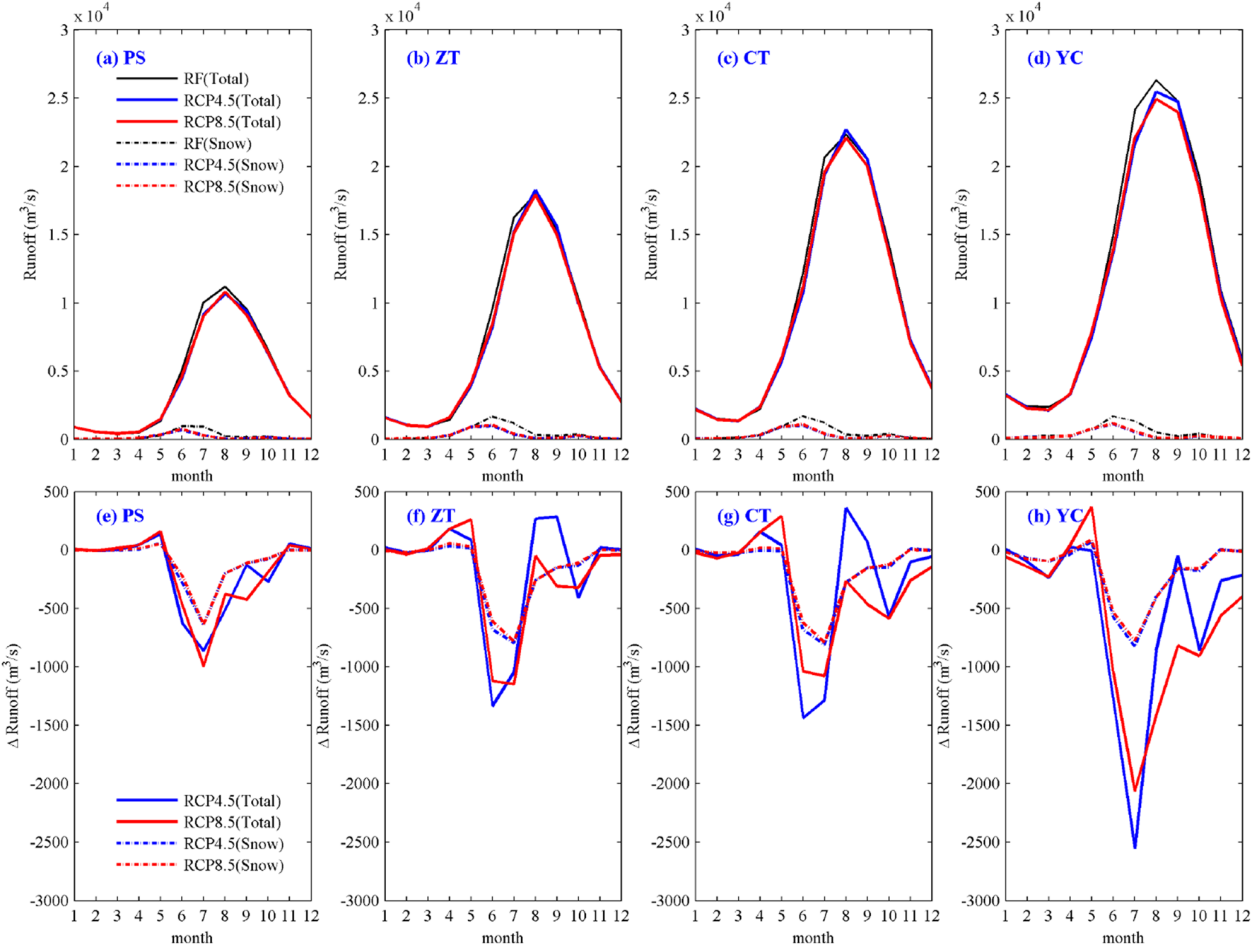
The multiyear average runoff process **(a–d)** of the R_t_ (solid line) and R_s_ (dashed line) for the PS, ZT, CT and YC stations under the RCP4.5 and RCP8.5 scenarios and the corresponding changes **(e–h)**. (The black color represents the reference period; the blue and red colors represent RCP4.5 and RCP8.5, respectively). The figure was generated by MATLAB2019a (https://www.mathworks.com/).

To study the characteristics of runoff changes under the near-future climate change in the UYRB, the average climate fields of R_CS and R_MPI were used as forcing data of the VIC hydrological model to simulate the near-future runoff process. Figure 10a–d shows the multiyear average runoff process of the total runoff (R_t_, solid line) and snowmelt runoff (R_s_, dashed line) for the PS, ZT, CT and YC stations under the RCPs (4.5 and 8.5), and the corresponding changes are presented (defined as the values in 2021–2050 minus those in 1971–2000). As shown in Fig. 10, compared with those in other seasons, the summer R_t_ and R_s_ will decrease more in the near-future. As shown in Fig. 10e–g, the decrease in R_t_ for the PS, ZT and CT stations located in the middle and upper reaches of the UYRB is largely due to the contribution of R_s_, whereas the decrease in R_t_ for the YC station at the outlet of the UYRB is less affected by R_s_ (Fig. 10 h). Table 10 shows the contribution of R_s_ to R_t_ in different seasons. In terms of the annual average, the R_s_ of the PS, ZT, CT and YC stations accounts for 5.9%, 6.0%, 4.8% and 4.1%, respectively, of the R_t_. The R_s_ contributes the most to the R_t_ in spring, with contributions of 17.8%, 19.5% and 14.6% for the PS, ZT and CT stations, respectively, while the contribution for YC is only 9.2%. The contribution of R_s_ to R_t_ is approximately 5–8% in summer but only approximately 1–2% in autumn and winter.

**Table 10:** Contribution of R_s_ to R_t_ in different seasons (unit: %).

	PS	ZT	CT	YC
Spring	17.8	19.5	14.6	9.2
Summer	8.1	7.2	5.9	5.4
Autumn	2.2	2.2	1.7	1.4
Winter	1.5	2.0	1.8	3.0
Average	5.9	6.0	4.8	4.1

Table 11 shows the multiyear average changes for the PS, ZT, CT and YC stations in different seasons under the RCPs (4.5 and 8.5) relative to the reference period. According to the results of the YC station at the outlet of the UYRB, R_t_ will decrease by approximately 4.4–5% in the near-future, while R_s_ will decrease by approximately 36.9–38.9%. The variation in R_t_ in different seasons is quite different, among which R_t_ decreases by approximately 6.9–7.2% in summer, 2.1–4.2% in autumn and 2.7–5.2% in winter. Note that the R_t_ in spring shows a slight opposite change in the near-future between the RCP4.5 and RCP8.5. Under the RCP4.5 scenario, the R_t_ in spring decrease by approximately -1.7%, whereas the R_t_ in spring increases by approximately 1.4% under the RCP8.5 scenario, which is related to the increase in R_s_ caused by climate warming. It can be seen from the spring R_s_ of other stations that the degree of increase of the R_s_ under the RCP8.5 scenario is significantly higher than that under the RCP4.5 scenario. In addition, the R_s_ of the PS station will increase significantly in spring, which is due to the melting of a large amount of snow cover in the upper reaches of the UYRB in winter, indicating that runoff changes in the source area of the Yangtze River are highly sensitive to climate warming.

**Table 11:** Multiyear average changes (unit: %) of the R_t_ and R_s_ for the PS, ZT, CT and YC stations in different seasons under the RCP4.5 and RCP8.5 scenarios relative to the reference period.

		PS		ZT		CT		YC	
		R_t_ (%)	R_s_ (%)	R_t_ (%)	R_s_ (%)	R_t_ (%)	R_s_ (%)	R_t_ (%)	R_s_ (%)
RCP4.5	Spring	8.3	15.2	4.2	3.6	1.7	− 2.7	− 1.7	− 5.0
	Summer	− 7.6	− 52.7	− 4.8	− 55.1	− 4.3	− 54.5	− 7.2	− 51.0
	Autumn	− 1.8	− 44.8	− 0.3	− 39.7	− 1.4	− 39.5	− 2.1	− 42.3
	Winter	0.4	− 14.5	0.1	− 24.4	− 1.3	− 24.4	− 2.7	− 26.1
	Average	− 4.2	− 42.1	− 2.3	− 38.7	− 2.5	− 38.9	− 4.4	− 38.9
RCP8.5	Spring	9.9	17.3	7.4	7.0	4.5	0.8	1.4	− 2.1
	Summer	− 7.0	− 49.9	− 5.3	− 52.6	− 4.3	− 52.1	− 6.9	− 48.5
	Autumn	− 3.0	− 44.2	− 2.2	− 37.8	− 3.1	− 37.7	− 4.2	− 40.8
	Winter	0.2	− 19.3	− 1.4	− 28.5	− 3.2	− 27.8	− 5.2	− 31.1
	Average	− 4.31	− 39.8	− 3.0	− 36.2	− 3.1	− 36.5	− 5.0	− 36.9

#### Spatial variation characteristics of hydrological elements

**Figure 11:**
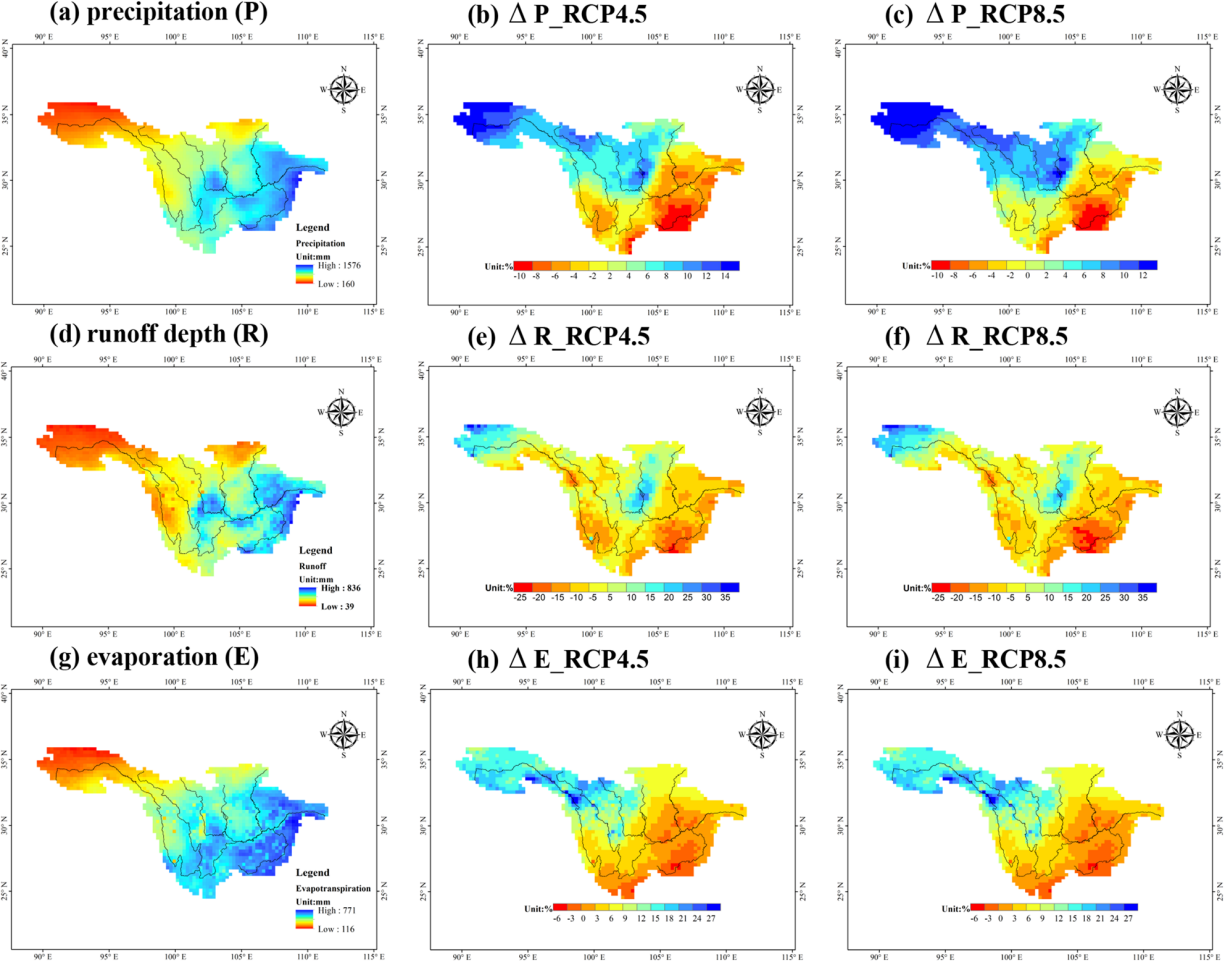
Spatial distribution of multiyear average precipitation (**a**, unit: mm), runoff depth (**d**, unit: mm) and evaporation (**g**, unit: mm) over the UYRB in the reference period and the corresponding changes (unit: %) under the RCP4.5 and RCP8.5 scenarios. The figure was generated by Arcmap 10.6 (https://www.esri.com).

Figure 11 shows the spatial distribution of the multiyear average precipitation, runoff depth and evaporation in the UYRB in the reference period and the corresponding changes under the RCPs (4.5 and 8.5). As shown in Fig. 11a, in the reference period, the southeastern part of the UYRB is the main area of precipitation, with annual precipitation exceeding 1000 mm, while the total annual precipitation in the northwest of the UYRB is usually approximately 200 mm. The spatial pattern of the multiyear average runoff depth in the reference period is nearly the same as that of precipitation, but the spatial distribution of multiyear average variation in the near-future differs between the southeast and northwest regions of the UYRB (Fig. 11d). According to the results of “Near-future climate change projected by RegCM4.5”, precipitation will decrease in the southeast area of the UYRB and increase in the northwest (Fig. 11b,c). The runoff depth will increase only in the source area of the YRB and the Minjiang River Basin in the middle of the UYRB under the RCPs (4.5 and 8.5), by approximately 15–25%, decreasing by approximately 5–25% in the other regions (Fig. 11e,f). The spatial distribution of evaporation changes is largely consistent with that of precipitation in the near-future. As shown in Fig. 11h–i, the evaporation in the northwestern UYRB will increase by approximately 20–30% in the near-future and decreases by approximately 5% in the southeastern UYRB.

#### Variation characteristics of extreme runoff

**Figure 12:**
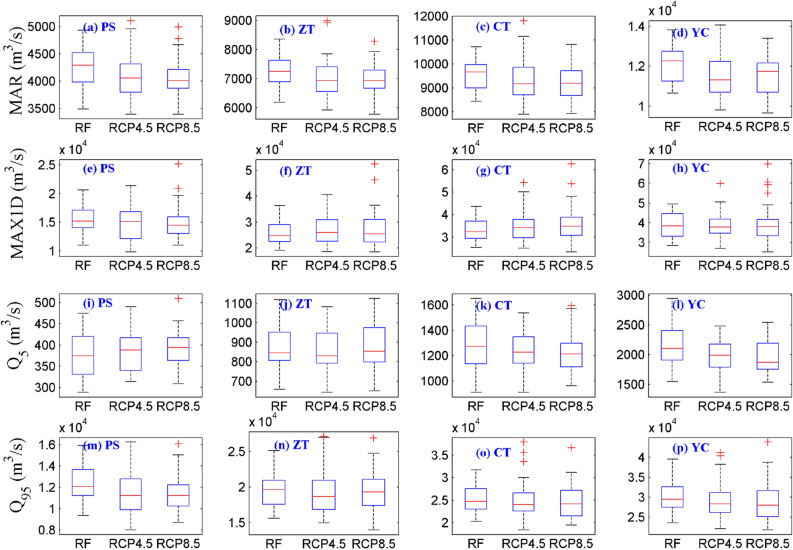
Box plots of the MAR **(a–d)**, MAX1D **(e–h)**, Q_5_ **(i–l)** and Q_95_ **(m–p)** for the PS, ZT, CT and YC stations for the reference period (RF) and future period (RCP4.5 and RCP8.5). The figure was generated by MATLAB2019a (https://www.mathworks.com/).

Figure 12 shows the box plots of the mean annual runoff (MAR), the maximum 1-day daily runoff (MAX1D), and the 5th and 95th percentile of daily runoff (Q_5_ and Q_95_) for the PS, ZT, CT and YC stations in the reference and near-future periods. To illustrate the extreme runoff changes in the UYRB, the YC station at the outlet of the basin is addressed here. As shown in Fig. 12d,h, compared with that in the reference period, the MAR of the YC will decrease under RCPs (4.5 and 8.5), whereas the MAX1D of the YC will not change significantly in the near-future. Compared with the reference period, both the Q_5_ and Q_95_ of the YC will decrease slightly in the near-future. The degree of decrease in Q_5_ and Q_95_ under the RCP8.5 scenario is slightly greater than that under the RCP4.5 scenario, but the degree of change mostly does not exceed the sample interval of the reference period. Note that there are many outliers of MAX1D and Q_95_ in the near-future that exceed the statistical interval of the reference period, especially under the RCP8.5 scenario.

## Discussions and conclusions

The UYRB is one of the regions in the Yangtze River Basin with frequent floods and is very sensitive to global warming. In this study, CSIRO-MK3.6.0 and MPI-ESM-MR were used to project the near-future climate change in the UYRB under RCPs (4.5 and 8.5), and meteorological elements from the dynamic downscaling results were revised using the QM method. Then, the revised climate forcing data were used to drive the hydrological model to simulate the hydrological process in the near-future over the UYRB. Finally, the spatiotemporal variation characteristics of runoff in the basin were analyzed under near-future climate changes. The main research conclusions are as follows:

According to the uncertainty analysis results, the depth of the second soil layer (*D*_2_) and the infiltration shape parameter (*B*) are the sensitive parameters in the VIC hydrological model. The VIC hydrological model has good simulation performance for daily and monthly runoff processes in the UYRB. The *NSE* is usually higher than 0.9 during the calibration and verification periods, and the *BIAS* is within ± 10%, indicating that the model is appropriate for the UYRB.

According to the statistical results of *D*, *P*_*95*_, *MRE*, *RMSE*, *SSE* and *CORR*, precipitation correction for the RCM results using the QM method based on a mixed distribution is better than that using the QM method based on a single distribution. The result indicates that the precipitation in the UYRB is not represented by a single precipitation pattern. In fact, the South Asian monsoon and East Asian monsoon have a strong influence on the precipitation in the YRB, so the mixed distribution can better describe the local complex precipitation pattern than the single distribution^15,16^. The results of Huang et al.^70^ confirm that the EASM and its subsystem SCSSM have much greater impact on precipitation in the YRB than on that in other basins in China. Among the five mixed distributions used in the study, the Gamma-GEV has the best performance in correcting precipitation over the UYRB and can effectively correct the obvious wet biases in simulated precipitation.

According to the revised results of R_CS and R_MPI, the eastern part of the UYRB will tend to be warm and dry relative to the reference period in the near-future, whereas the western part of the basin will tend to be warm and wet. The precipitation will generally decrease at a rate of 19.05–19.25 mm/10 a, but the trend is not obvious. The T_2m_ will increase significantly at a rate of 0.38–0.52 °C/10 a, and the temperature will rise by approximately 1.5 °C in the mid-twenty-first century. Huang et al.^70^ showed that the summer precipitation in the UYRB is predicted to decrease significantly in the mid-21th century, which is consistent with the results of Cao et al., Deng et al. and Wang et al.^22,30,71^. Moreover, as the temperature rises, the difference in precipitation between the northwest and southeast of the basin will increase, and the risk of flood disaster caused by high-intensity precipitation in the western and central regions may increase^70^.

The contribution of snowmelt runoff to the annual runoff of the UYRB is only approximately 4%, and the contribution can reach approximately 9.2% in the spring. Affected by climate warming, snowmelt runoff will decrease by approximately 36–39% in the near-future, while annual runoff will decrease by approximately 4.1–5%, and extreme runoff will slightly decrease. Regarding the spatial changes in runoff depth, the areas of decreased runoff are concentrated in the southeast of the basin. The decrease in precipitation is the direct factor leading to the decrease in runoff depth in the southeast of the basin, while the decrease in runoff depth in the northwest is mainly affected by the increase in evaporation. These findings are consistent with previous studies on the impacts of climate change in the UYRB^22,30,72,73^. In addition, due to climate warming, more rainfall than snowfall may increase the risk of summer droughts or spring floods in the snow-covered basin, and this risk will increase as the rate of temperature rise increases^30,74,75^. The temperature increase in winter and spring may cause the melting of glaciers and snow at the source of the Yangtze River, where most of the glacier surface is located, and lead to a large flow increase in April^30^. From May to September, the water flow decreases, which may exacerbate the crisis of water shortage in the UYRB during the flood season.

However, the findings of this study are not completely consistent with some of the findings of Gu et al.^29^ and Su et al.^26^ Due to study differences in source data, bias correction methods, global climate models, hydrological model structure, model parameterization, reference period, comparison period, and emission scenarios, which may introduce great uncertainty in the assessment of the impact of future climate change, inconsistent results among studies may occur^76^. For example, Gu et al.^29^ used the gamma distribution to revise the precipitation from the RegCM4; however, the present study revealed that a single distribution (such as the gamma distribution) was not the best choice. Compared with the mixed distribution, a single distribution will yield a large amount of wet bias in the revised precipitation, resulting in excessive precipitation, as confirmed by Shin et al.^45^ Some studies have indicated that the uncertainty generated by the use of corrected forcing data in hydrological response studies may be of the same order of magnitude as that in the GCMs and hydrological models^77,78^. Previous studies have confirmed that the main source of uncertainty in future runoff forecasts in the UYRB is related to the choice of climate forcing (GCMs and RCPs) and that hydrological models paly only a secondary role^26,79^. However, in most previous studies, empirical manual trial calculation has been adopted to obtain the hydrological model parameters^22,29^, whereas in this study, the GLUE was adopted to select the optimal parameter group from a large number of sample parameters.

In this study, R_CS and R_MPI were used in the regional hydrological and climate projection of the UYRB, which was helpful to estimate future climate-related risks. However, it is still necessary to combine the results of more RCMs or GCMs to objectively project the climate change characteristics of the UYRB. In addition, the spatial and temporal variability of runoff may be influenced by various anthropogenic activities (e.g., irrigation, land-use change, reservoir operation), which were not considered in this study. This study aimed to provide overall and regional trends of the UYRB under a specific model, scenario, and method, rather than make accurate projections for a specific location.

## Supplementary Information

Supplementary Information.
