# Supplementary material for: Hydrological projections in the upper reaches of the Yangtze River Basin from 2020 to 2050
Source: Sci Rep. 2021 May 6;11:9720. doi: 10.1038/s41598-021-88135-5 (PMC8102517; doi:10.1038/s41598-021-88135-5)
Supplement: Supplementary file 1 — Supplementary Information. [file 41598_2021_88135_MOESM1_ESM.pdf]

Electronic Supplement Material

Hydrological projections in the upper reaches of the Yangtze River Basin from 2020 to 2050

Ya Huang[a,b,c,d*], Weihua Xiao[a], Baodeng Hou[a], Yuyan Zhou[a], Guibing Hou[c], Ling Yi[c], Hao Cui[a]

a. State Key Laboratory of Simulation and Regulation of Water Cycle in River Catchment, China Institute of Water Resources and Hydropower Research, Beijing 100038, China.
b. College of Oceanography, Hohai University, Nanjing 210098, China;
c. China Water Resources Pearl River Planning, Surveying & Designing Co., Ltd., Guangzhou 510610, China.
d. Global Institute for Water Security, University of Saskatchewan, Saskatoon, SK, Canada.

Corresponding authors: Ya Huang

Address: Zhanyi Road 19#, Guangzhou, 510610, China

Email: hygccw@163.com

Tel:0086-020-87117060

Table S1 VIC model parameter configuration.

Parameter	Units	Description	Range	Fitted value
B	-	Variable infiltration curve parameter	0~0.6	0.59
D_s	fraction	Fraction of Dsmax where nonlinear baseflow begins	0~1	0.06
D_{smax}	mm/d	Maximum velocity of the baseflow	0~30	20.1
W_s	fraction	Fraction of the maximum soil moisture where nonlinear baseflow occurs	0.5~1	0.5
D_2	m	Depth of the second soil layer	0.1~1.5	0.1
D_3	m	Depth of the third soil layer	0.1~1.5	0.35

Table S2 Simulation performance of daily runoff processes in the wet year and the dry year.

**Year**	**Stations**	***NSE***	***BIAS* (%)**
Wet year (1981)	PS	0.90	-5.56
	ZT	0.93	-9.33
	CT	0.92	-0.55
	YC	0.90	-0.41
Dry year (1994)	PS	0.68	13.52
	ZT	0.85	-0.45
	CT	0.86	0.32
	YC	0.81	0.33

Table S3 Changes and trends of T_{max} from R_CS and R_MPI under the RCP4.5 and RCP8.5 scenarios in different periods.

Period	R_CS		R_MPI		Average	
	RCP4.5	RCP8.5	RCP4.5	RCP8.5	RCP4.5	RCP8.5
2021-2030	1.172	1.419	1.089	1.053	1.130	1.236
2031-2040	1.632	1.575	1.321	1.385	1.476	1.480
2041-2050	2.118	2.468	1.761	1.775	1.940	2.121
Mean	1.641	1.820	1.390	1.405	1.516	1.612
Trend (℃/10 a)	0.40^{**}	0.52^{**}	0.22^{*}	0.21^{*}	0.31^{*}	0.42^{*}

*indicates a significant value at the 0.01 level
**indicates a significant value at the 0.05 level

Table S4 Changes and trends of T_{min} from R_CS and R_MPI under the RCP4.5 and RCP8.5 scenarios in different periods.

Period	R_CS		R_MPI		Average	
	**RCP4.5**	**RCP8.5**	**RCP4.5**	**RCP8.5**	**RCP4.5**	**RCP8.5**
2021-2030	1.103	1.307	1.000	0.984	1.051	1.145
2031-2040	1.633	1.581	1.143	1.210	1.388	1.395
2041-2050	2.039	2.346	1.694	1.728	1.866	2.037
mean	1.592	1.745	1.279	1.307	1.435	1.526
Trend (℃/10 a)	0.4**	0.52**	0.22**	0.21**	0.31**	0.42**

*indicates a significant value at the 0.01 level
**indicates a significant value at the 0.05 level

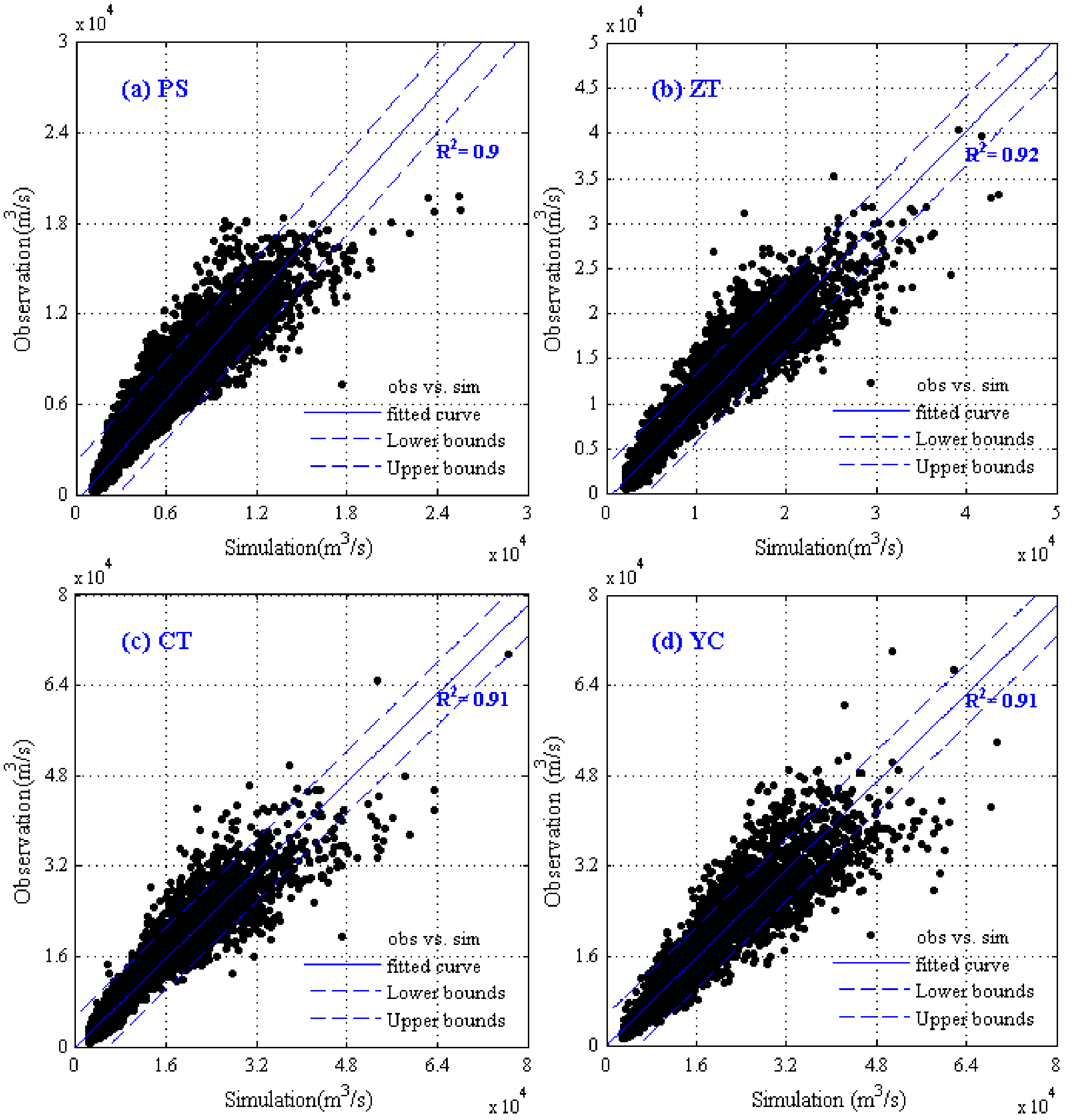

Fig. S1 Scatter plots of simulated and observed daily runoff during the verification period (**a** PS station; **b** ZT station; **c** CT station; **d** YC station). The figure was generated by MATLAB2019a (https://www.mathworks.com/).

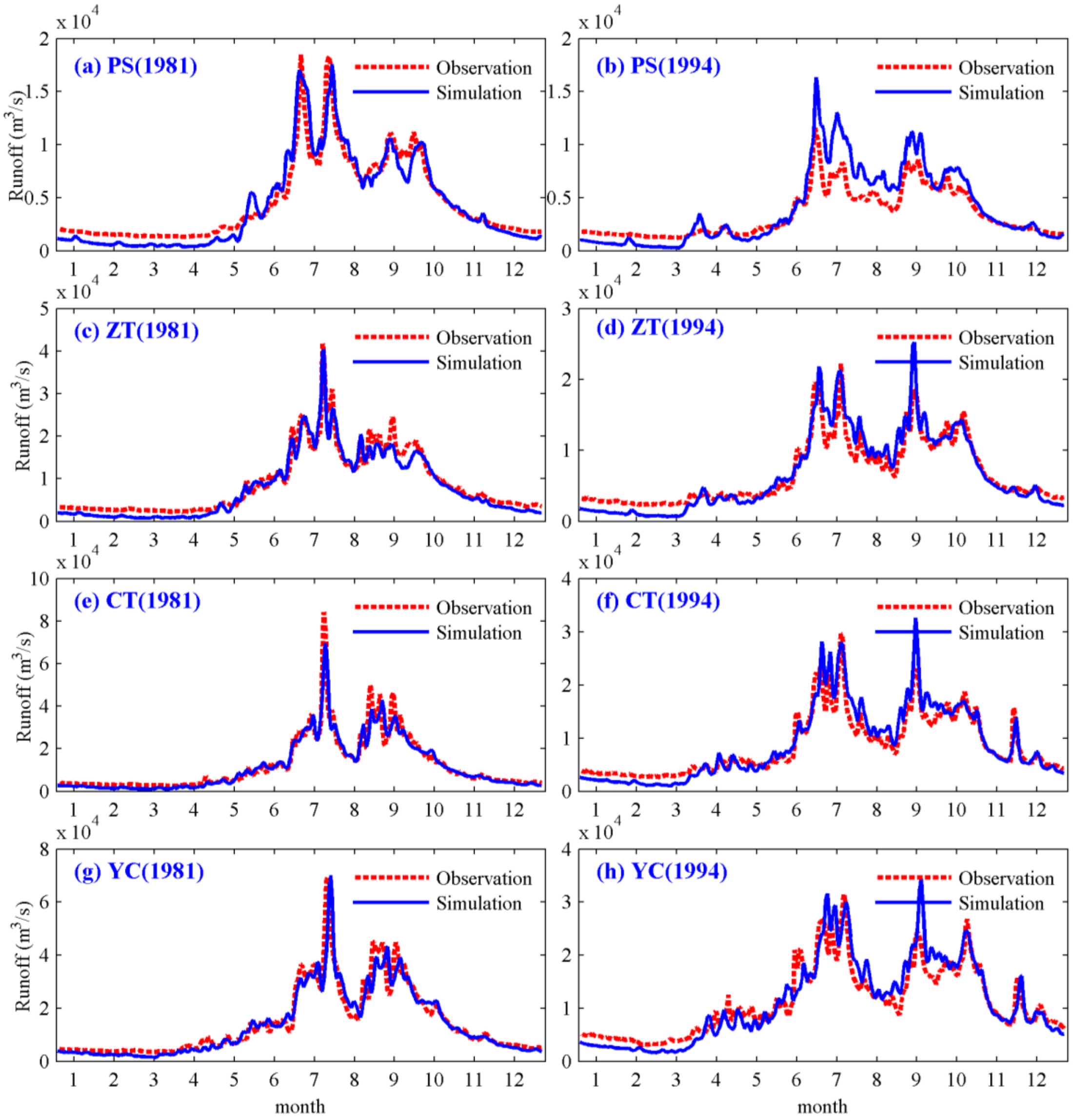

Fig. S2 Daily simulated and observed runoff processes in the wet year (1981, left panels) and the dry year (1994, right panels). The figure was generated by MATLAB2019a (https://www.mathworks.com/).

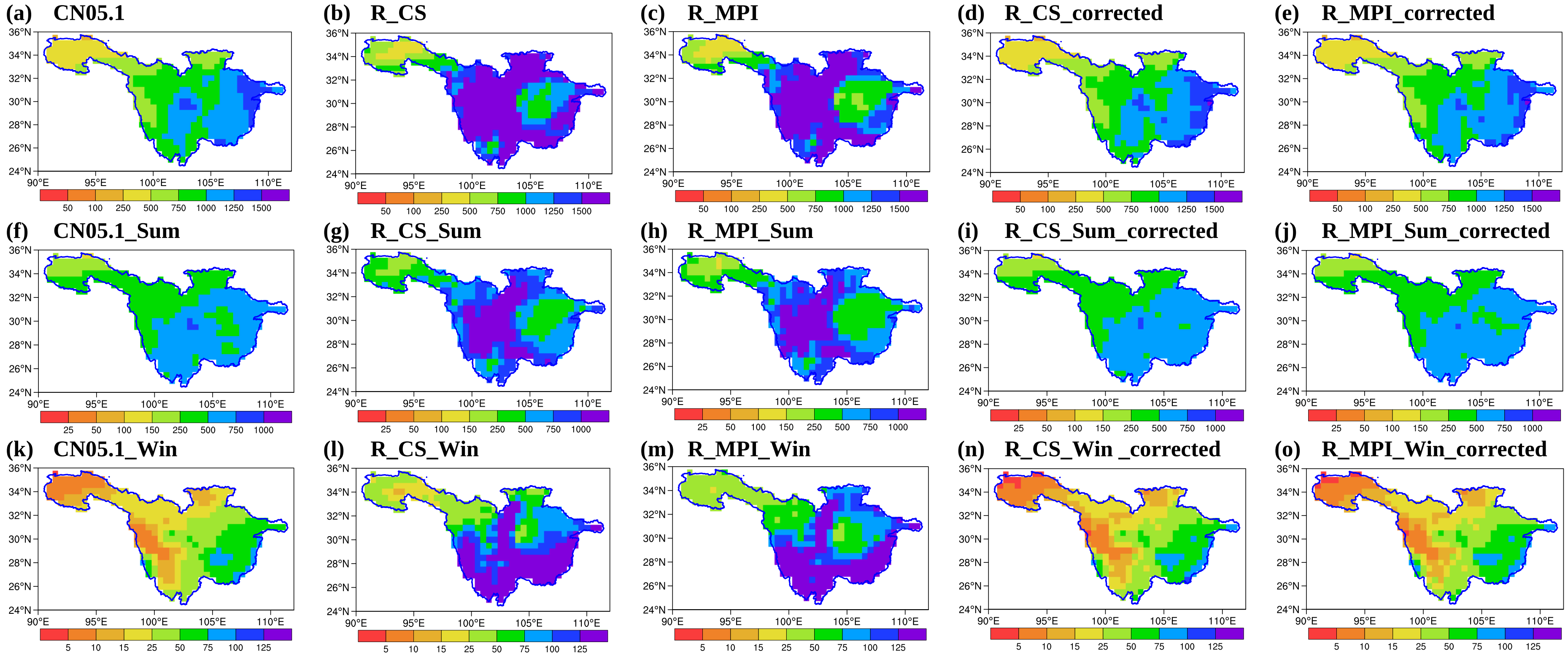

Fig. S3 Spatial distribution of the multiyear average precipitation (unit: mm) for the reference period (1971–2000) over the UYRB. [**a** CN05.1 data, **b** precipitation before the revision from the R_CS, **c** precipitation before the revision from the R_MPI, **d** precipitation after the revision from the R_CS, **e** precipitation after the revision from the R_MPI, **f-j** and **k-o** are the same as **a-e** but for the summer and winter, respectively.] The figure was prepared using The NCAR Command Language version 6.5.0. (https://doi.org/10.5065/D6WD3XH5).

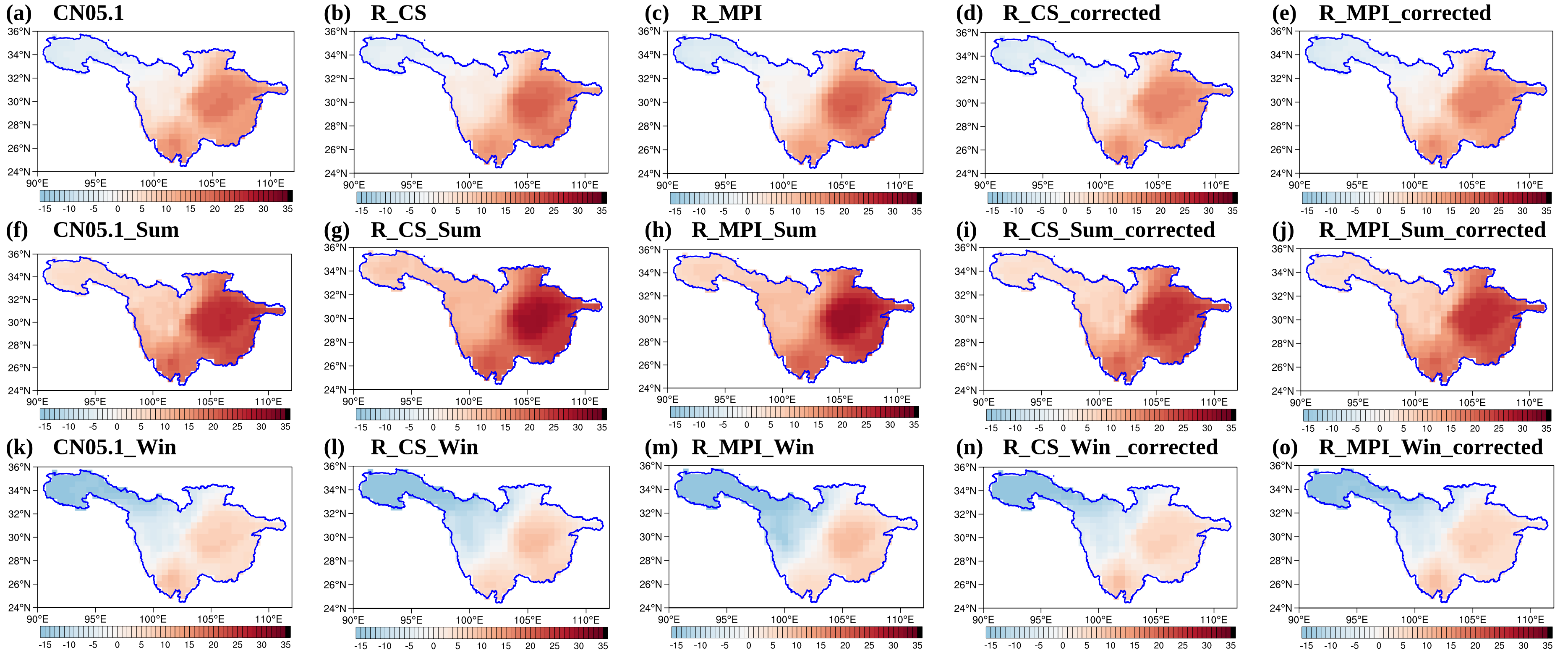

Fig. S4 Spatial distribution of the multiyear average T_{2m} (unit: ℃) for the reference period (1971–2000) over the UYRB. [**a** CN05.1 data, **b** T_{2m} before the revision from the R_CS, **c** T_{2m} before the revision from the R_MPI, **d** T_{2m} after the revision from the R_CS, **e** T_{2m} after the revision from the R_MPI, **f-j** and **k-o** are the same as **a-e** but for the summer and winter, respectively.] The figure was prepared using The NCAR Command Language version 6.5.0. (https://doi.org/10.5065/D6WD3XH5).

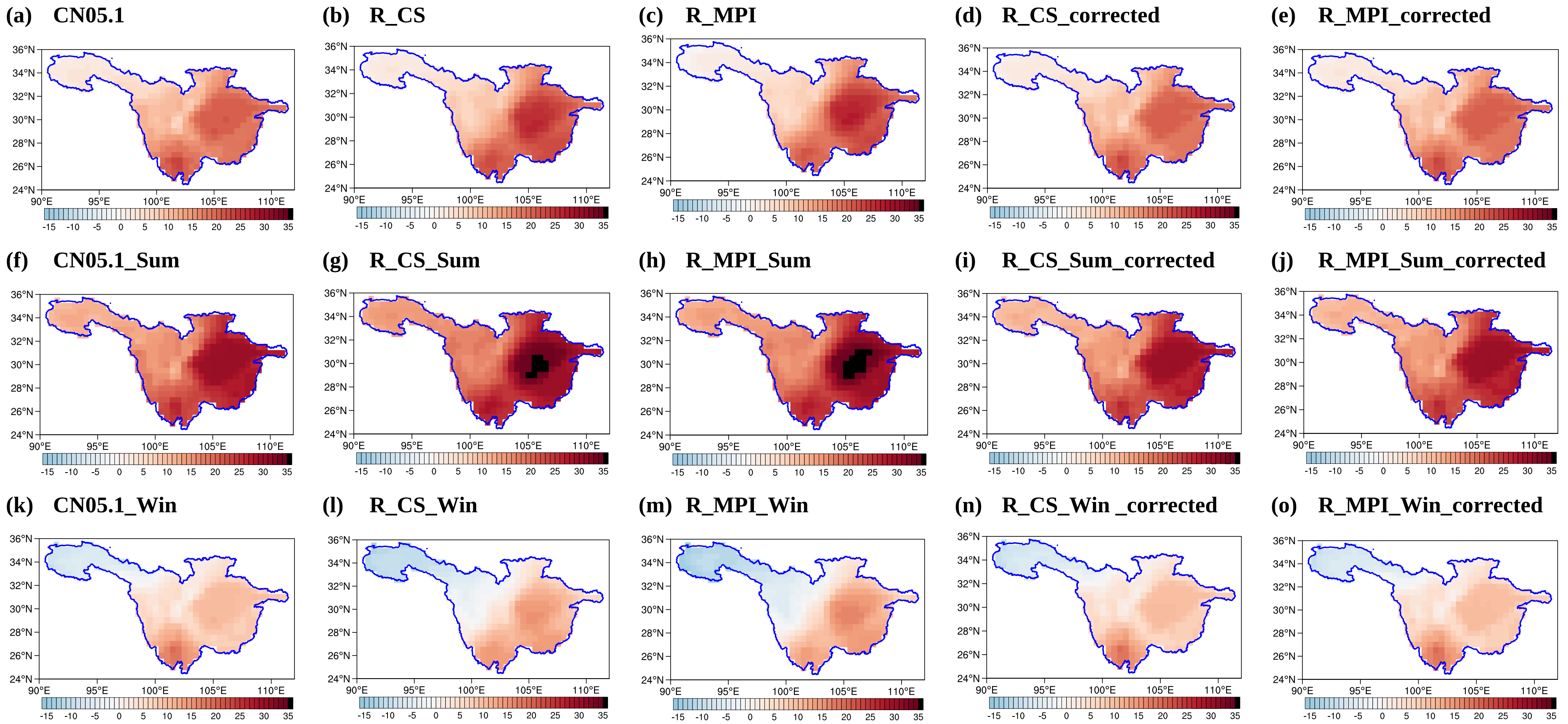

Fig. S5 Spatial distribution of the multiyear average T_{max} (unit: ℃) for the reference period (1971–2000) over the UYRB. [**a** CN05.1 data, **b** T_{max} before the revision from the R_CS, **c** T_{max} before the revision from the R_MPI, **d** T_{max} after the revision from the R_CS, **e** T_{max} after the revision from the R_MPI, **f-j** and **k-o** are the same as **a-e** but for the summer and winter, respectively.] The figure was prepared using The NCAR Command Language version 6.5.0. (https://doi.org/10.5065/D6WD3XH5).

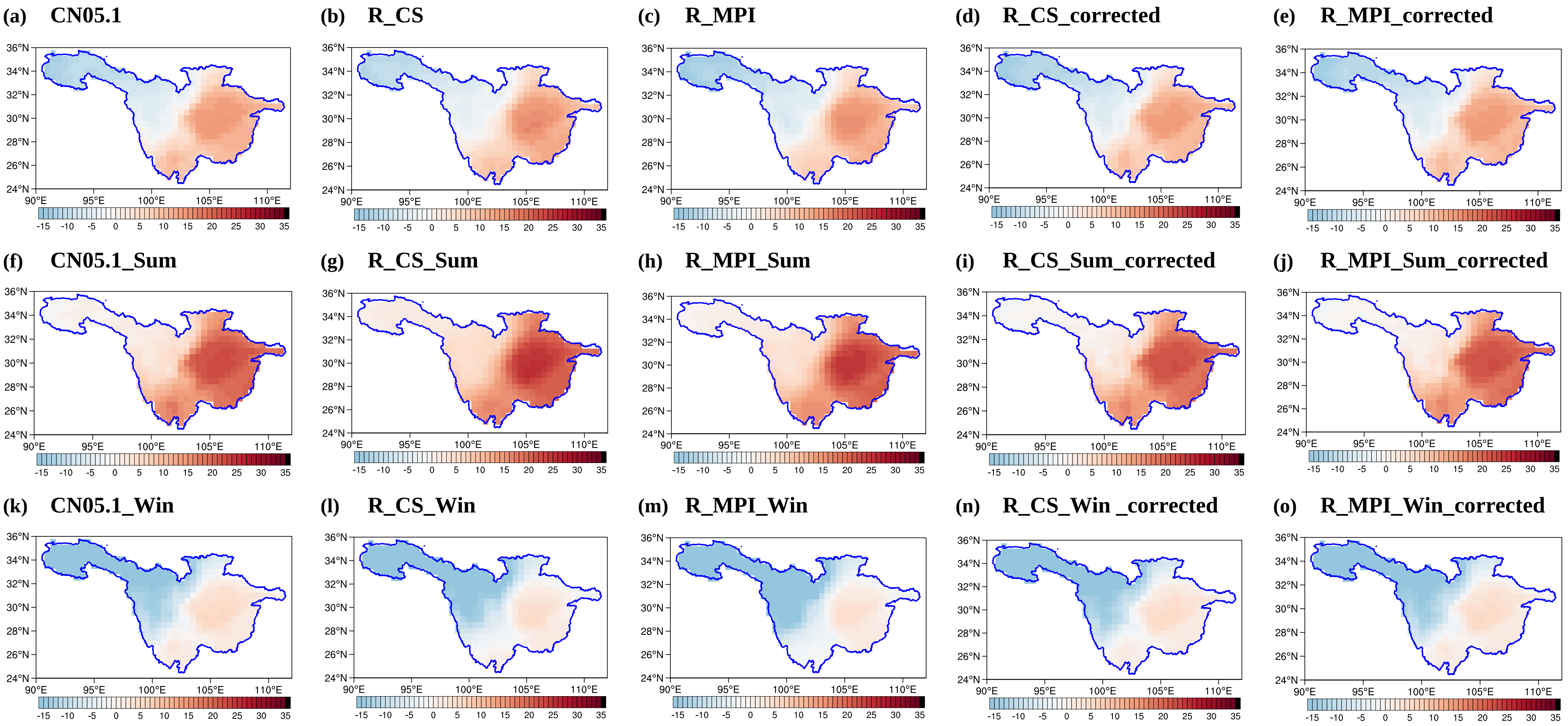

Fig. S6 Spatial distribution of the multiyear average T_{min} (unit: °C) for the reference period (1971–2000) over the UYRB. [**a** CN05.1 data, **b** T_{min} before the revision from the R_CS, **c** T_{min} before the revision from the R_MPI, **d** T_{min} after the revision from the R_CS, **e** T_{min} after the revision from the R_MPI, **f-j** and **k-o** are the same as **a-e** but for the summer and winter, respectively.] The figure was prepared using The NCAR Command Language version 6.5.0. (https://doi.org/10.5065/D6WD3XH5).

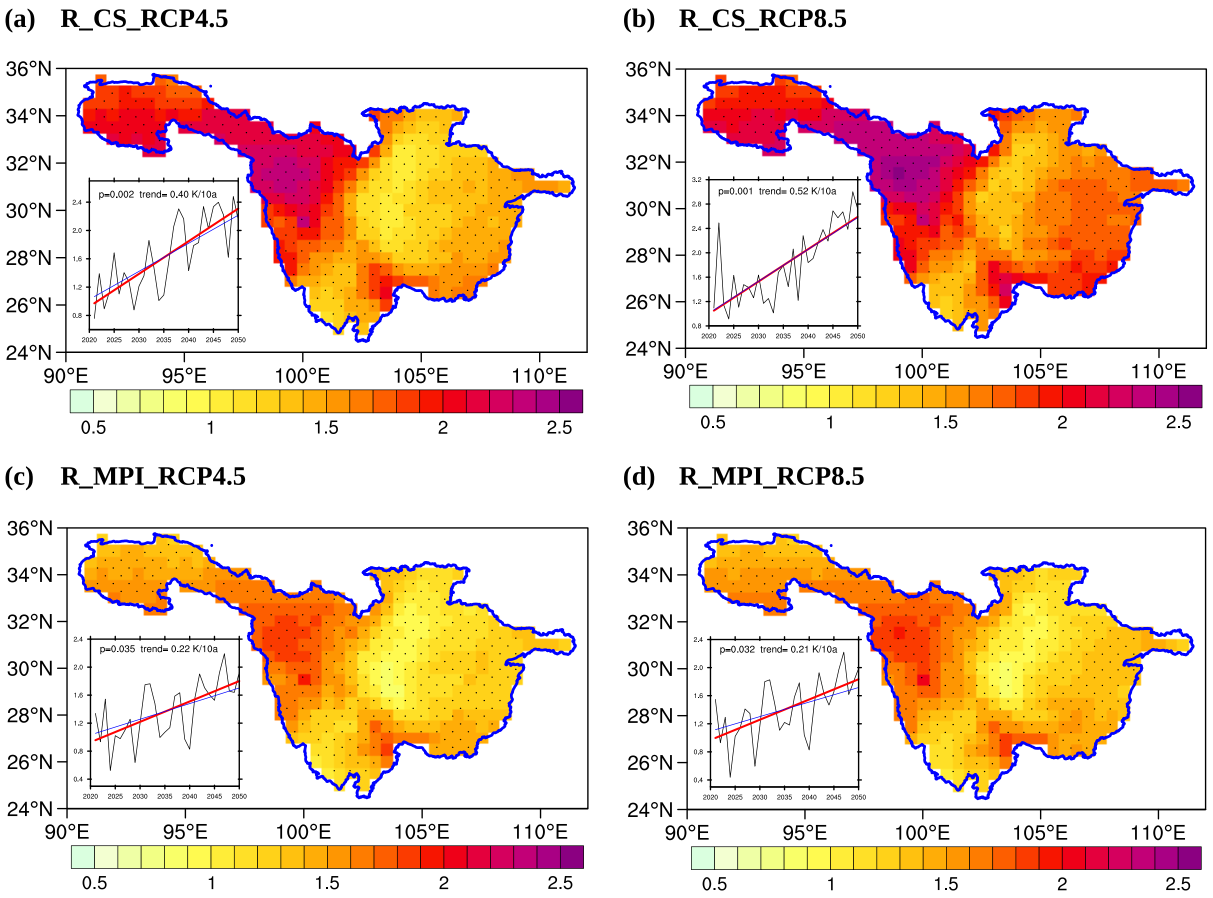

Fig. S7 Multiyear average changes (unit: ℃) in T_{max} over the UYRB under the RCP4.5 and RCP8.5 scenarios compared to the reference period (1971-2000). The black dots denote differences that are statistically significant at a significance level of 95% based on Student's t-test. The rectangle indicates the interannual variation trend of maximum air temperature anomalies (unit: ℃). The figure was prepared using The NCAR Command Language version 6.5.0. (https://doi.org/10.5065/D6WD3XH5).

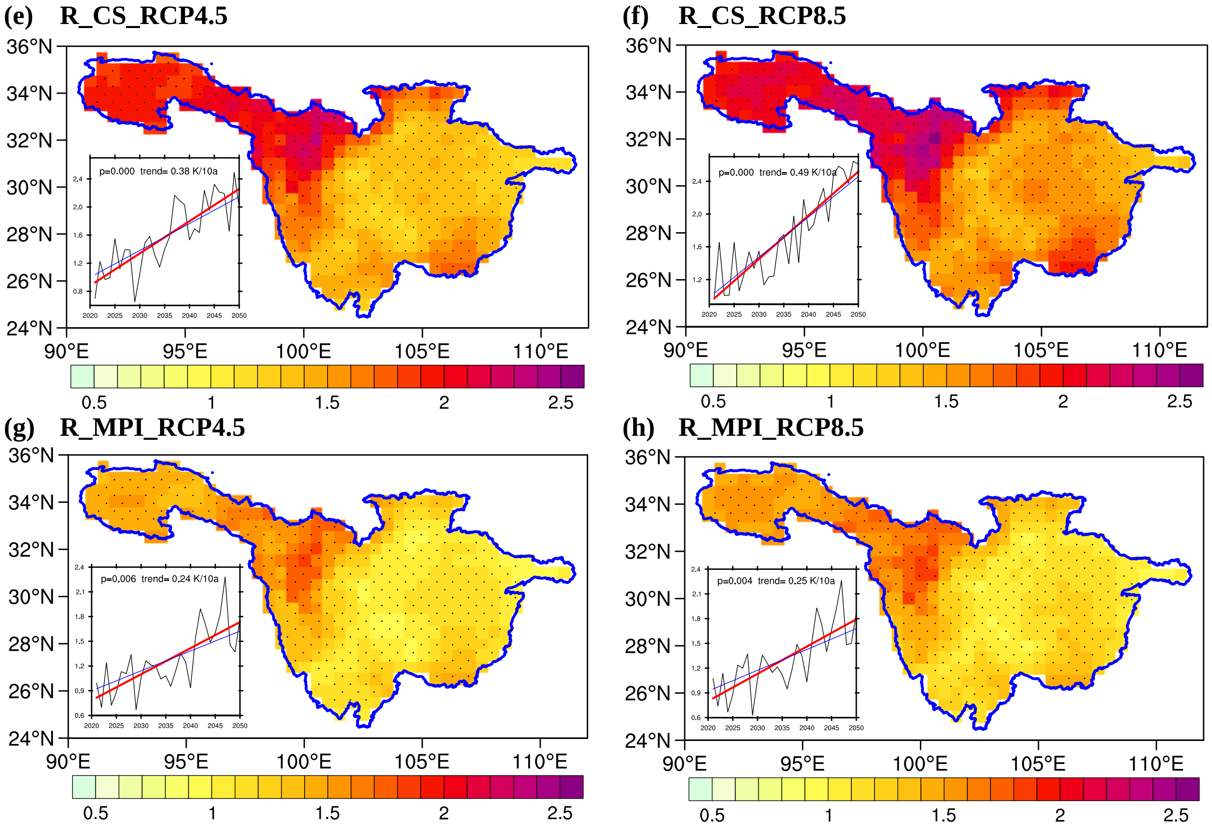

Fig. S8 Multiyear average changes (unit: ℃) in T_{min} over the UYRB under the RCP4.5 and RCP8.5 scenarios compared to the reference period (1971-2000). The black dots denote differences that are statistically significant at a significance level of 95% based on Student's t-test. The rectangle indicates the interannual variation trend of minimum air temperature anomalies (unit: ℃). The figure was prepared using The NCAR Command Language version 6.5.0. (https://doi.org/10.5065/D6WD3XH5).
